# Exercise-induced myokines in metabolic regulation: mechanisms, mimetics, and translational potential

**DOI:** 10.1007/s12272-026-01624-x

**Published:** 2026-06-19

**Authors:** Muhammad Arif Aslam, Jongmin Lee, T. Scott Bowen, Joo Young Huh

**Affiliations:** 1 College of Pharmacy, Chonnam National University, Gwangju, Republic of Korea; 2 Department of Global Innovative Drugs, The Graduate School of Chung-Ang University, Seoul, Republic of Korea; 3 School of Biomedical Sciences, Faculty of Biological Sciences, University of Leeds, Leeds, UK; 4 College of Pharmacy, Chung-Ang University, 84 Heukseok-ro, Dongjak-gu, Seoul, 06974 Republic of Korea

**Keywords:** Exercise, Myokines, Metabolism, Skeletal muscle, Muscle–organ crosstalk

## Abstract

Skeletal muscle, once regarded solely as a contractile tissue, is now recognized as a dynamic endocrine organ that secretes exercise-induced myokines—bioactive peptides with autocrine, paracrine, and endocrine functions. These myokines coordinate systemic energy homeostasis by regulating glucose and lipid metabolism, mitochondrial function, inflammation, and interorgan communication. Building on our previous review published in 2018, this review synthesizes major advances in exercise-induced myokines within an evidence-based framework considering mechanistic support and translational relevance. We highlight both well-established and emerging myokines, including interleukin-6 (IL-6), irisin, myostatin, growth differentiation factor 11 (GDF11), IL-15, brain-derived neurotrophic factor (BDNF), meteorin-like (METRNL), secreted protein acidic and rich in cysteine (SPARC), fibroblast growth factor 21 (FGF21), β-aminoisobutyric acid (BAIBA), leukemia inhibitory factor (LIF), apelin, and musclin, and discuss their roles across major target tissues including skeletal muscle, liver, adipose tissue, and bone. We also summarize natural and synthetic compounds reported to modulate myokine expression, secretion, or activity, and discuss the opportunities and current limitations of targeting myokine pathways. Although several myokine axes show therapeutic promise, the current literature indicates substantial heterogeneity in causal evidence, receptor or target certainty, and translational readiness. These insights support a more selective view of myokines as biologically heterogeneous mediators of muscle–organ crosstalk and provide a framework for mechanism-based therapeutic development in metabolic disease.

## Introduction

Exercise influences gene expression, modulates cell signaling pathways, enhances organ system function, and ultimately benefits whole-body physiology (Chow et al. 2022). In contrast, a sedentary lifestyle promotes metabolic, cardiovascular, hormonal, and musculoskeletal dysfunctions, thereby increasing the risk of chronic diseases and all-cause mortality (Park et al. 2020). Consequently, investigating various exercise modalities enables the identification of novel biomarkers and therapeutic targets relevant to conditions driven by physical inactivity. Despite substantial progress over several decades, elucidating the complex biological mechanisms activated by exercise remains challenging as the responses involve tightly coordinated interactions across multiple tissues and physiological systems. Although considerable research has improved our understanding of the molecular and cellular effects of acute and chronic exercise, many mechanistic aspects remain unresolved.

As the largest organ in the body, comprising approximately 40% of the total body mass, skeletal muscles play a central role in metabolic regulation (Das et al. 2020). Exercise elicits molecular responses in skeletal muscles and chronic training promotes sustained phenotypic adaptation. These adaptations include improved mitochondrial function, enhanced glucose and lipid metabolism, and increased oxidative capacity, particularly during aerobic exercise (Yu et al. 2016; Heo et al. 2021). Resistance training stimulates muscle hypertrophy and strength through signaling pathways, such as mTOR and p70S6K, and is further modulated by anabolic hormones, including IGF-1 (Feng et al. 2022). Beyond its mechanical and metabolic contributions, skeletal muscle is now widely recognized as a pivotal endocrine organ, which was first proposed more than five decades ago based on observations that muscle contraction can influence distant organ function (Goldstein 1961). Skeletal muscles are well known to secrete hundreds of cytokines and peptides, collectively termed myokines, in response to exercise (Severinsen and Pedersen 2020; Chen et al. 2021). Myokines have reshaped modern exercise physiology by demonstrating that skeletal muscles communicate with distant tissues, such as the liver, brain, bone, and adipose tissue, through autocrine, paracrine, and endocrine signaling, thereby regulating both muscle adaptation and systemic metabolic health. Thus, understanding these muscle-derived factors offers critical insights into how exercise maintains systemic homeostasis.

**Fig. 1:**
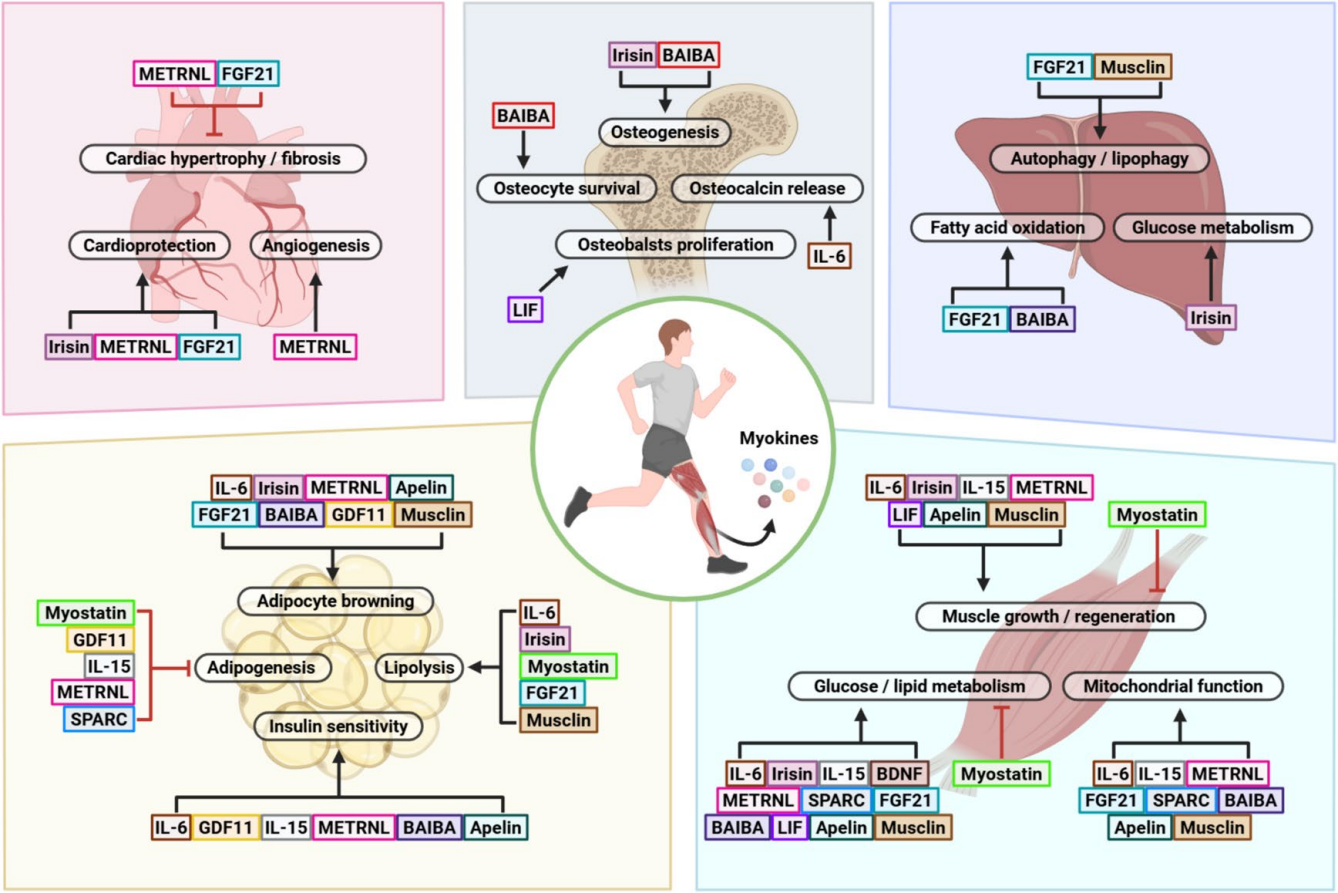
Overview of the major exercise-induced myokines and their principal roles in inter-organ metabolic communication. Skeletal muscle-derived factors influence the heart, bone, liver, adipose tissue, and skeletal muscle through organ-specific interconnected functional networks. *APJ* apelin receptor, *BAIBA* β-aminoisobutyric acid, *BDNF* brain-derived neurotrophic factor, *FGF21* fibroblast growth factor 21, *FNDC5* fibronectin type III domain-containing protein 5, *GDF11* growth differentiation factor 11, *IL-6* interleukin-6, *IL-15* interleukin-15, *LIF* leukemia inhibitory factor, *METRNL* meteorin-like, *SPARC* secreted protein acidic and rich in cysteine

Building on our earlier review of exercise-induced myokines and their roles in metabolic regulation (Huh 2018), the present article revisits this field in light of the major advances made over the past several years. Since that initial contribution, an expanding body of work has refined our understanding of how myokines act within skeletal muscle and across distant organs. This review therefore provides an updated overview of exercise-responsive myokines as mediators of interorgan communication and systemic metabolic adaptation, with emphasis on the molecular pathways linking skeletal muscle to key metabolic organs (Fig. 1).

To provide both mechanistic synthesis and critical perspective, the review first evaluates major myokines by evidence strength, receptor definition, and degree of human support, distinguishing well-supported mediators from less-established candidates (Tables 1 and 2). It then summarizes natural and synthetic compounds modulating myokine expression and secretion, highlighting opportunities to mimic or enhance muscle–organ signaling (Table 3). The review also discusses the therapeutic potential of targeting myokine pathways, current challenges, and future priorities in the field. To provide a structured framework, we group the myokines reviewed here into three tiers according to criteria weighted hierarchically rather than equally: demonstrable human interventional or causal evidence is the decisive criterion and defines Tier 1; receptor- or target-defined mediators supported by consistent but still largely correlative human data constitute Tier 2; and exploratory or hypothesis-generating candidates whose mediator status, receptor identity, or assay validity remains unresolved constitute Tier 3. Pharmacological tractability and clinical-development status rank factors within a tier rather than assign it. Accordingly, Tier 1 comprises interleukin (IL)-6, myostatin/ActRII, fibroblast growth factor 21 (FGF21), and apelin/APJ. Tier 2 comprises irisin/FNDC5, IL-15, meteorin-like (METRNL), and brain-derived neurotrophic factor (BDNF; acting predominantly as a local autocrine/paracrine mediator). Tier 3 comprises growth differentiation factor 11 (GDF11), secreted protein acidic and rich in cysteine (SPARC), β-aminoisobutyric acid (BAIBA), leukemia inhibitory factor (LIF; as an endocrine factor), and musclin, together with several less-characterized factors discussed at the end of the next section. These tier assignments are summarized in Table 2 and revisited in the translational discussion below.

**Table 1:** Myokines regulated by exercise: target organs and metabolic effects

Myokine	Target organ	Metabolic effects and mechanisms	References
IL-6	Muscle	↑ AMPK → ↑ GLUT4 and lipolysis	Kelly et al. (2009); Ikeda et al. (2016)
		↑ Satellite cell hypertrophy via STAT3	Serrano et al. (2008)
		REV-ERBα-dependent mitochondrial remodeling and autophagy	Pinto et al. (2022)
		Piezo1↓ → KLF15–IL-6 axis in immobilization-induced atrophy	Hirata et al. (2022)
		Chronic elevation: ↑ gp130 → ↓ mitochondrial function, ↑ fatigue	Vanderveen et al. (2019)
	Adipose tissue	↑ UCP1 browning	Knudsen et al. (2014)
		↑ Lipolysis and fat oxidation	Wedell-Neergaard et al. (2019)
		↑ Glyceroneogenesis and anti-inflammatory responses in visceral fat	Bertholdt et al. ( 2020)
	Bone	Osteoblast IL-6R → RANKL/osteoclastogenesis → ↑ osteocalcin release	Chowdhury et al. (2020)
	Gut	↓ Gastric emptying → ↓ postprandial glycemia (GLP-1-independent)	Lang Lehrskov et al. (2018)
Irisin/FNDC5	Muscle	↑ AMPK → ↑ glucose and lipid metabolism	Huh et al. (2014); Lee et al. (2015)
		↑ IGF-1/Akt/ERK → ↓ dexamethasone-induced atrophy	Chang and Kong (2020)
		Suppresses MuRF-1 and Atrogin-1 → ↓ sarcopenia	Guo et al. (2023)
		PI3K/Akt–Nrf2 → ↓ fibrosis and oxidative stress	Wu et al. (2023)
		FOXO3 and ALCAT1 modulation in CKD/MI-induced wasting	Wang et al. (2024); Ren et al. (2022)
	Adipose tissue	↑ p38 MAPK and ERK1/2 → UCP1-mediated browning	Zhang et al. (2014)
		↑ cAMP–PKA–perilipin/HSL → lipolysis	Xiong et al. (2015)
		CD81-integrin-FAK axis in beige progenitor cells	Oguri et al. (2020)
	Liver	↑ Glycogen synthesis (PI3K/Akt) ↓ gluconeogenesis	Liu et al. (2019a)
		↓ Cholesterol synthesis (AMPK–SREBP2)	Tang et al. (2016)
	Bone	Integrin αVβ5-ERK/STAT and BMP/Smad → ↑ osteogenesis	Xue et al. (2022)
		Sex-specific effects on bone mass and osteocyte transcriptome	Shimonty et al. (2024)
	Heart	Integrin αVβ5 → ↑ Akt → cardioprotection in diabetic cardiomyopathy	Lin et al. (2021)
	Kidney	Integrin αVβ5 → ↓ mtDNA leakage, ↓ cGAS–STING inflammation in acute kidney injury	Peng et al. (2025)
Myostatin	Muscle	Myostatin activation: ActRIIB/ALK4/5 → Smad2/3 → ↓ muscle growth and regeneration; non-canonical pathways: Wnt, Notch, MAPK, PI3K-Akt → wasting and atrophy; extracellular antagonism by follistatin, FSTL-3, and GASP-1	Mcpherron et al. (1997); Rodgers and Ward (2022); Lee (2021)
		Myostatin inhibition → ↑ GLUT1/4 → ↑ glucose uptake and insulin sensitivity; improved FA metabolism and mitochondrial gene translation (independent of hypertrophy)	Abati et al. (2022); Eilers et al. (2020)
	Adipose tissue	↑ Lipolysis and mitochondrial lipid oxidation → ↓ obesity and insulin resistance	Zhang et al. (2012)
		miR-124-3p targets glucocorticoid receptor → ↓ adipogenesis	Liu et al. (2019b)
GDF11	Muscle	ActRII-like → Smad2/3 → FOXO1 → ubiquitin–proteasome and autophagy pathways	Zimmers et al. (2017); Honda et al. (2022)
		Context-dependent: high doses → atrophy (LC3-II/I ↑), low doses or propeptide inhibition → regeneration and hypertrophy	Sinha et al. (2014); Egerman et al. (2015); Jin et al. (2019)
	Adipose tissue	↓ White adipocyte size and ↑ glucose uptake in mature adipocytes (PI3K/Akt, AMPK)	Frohlich et al. (2022); Lu et al. (2019)
		↓ Adipogenesis via Wnt/β-catenin and Smad2/3	Frohlich et al. (2022); Lin et al. (2023)
		↑ Energy expenditure via TGF-β/Smad2	Lu et al. (2019)
IL-15	Muscle	JAK3/STAT3 → ↑ glucose uptake	Krolopp et al. (2016)
		↑ GLUT4 translocation and AMPK phosphorylation	Fujimoto et al. (2019)
		PPARδ-dependent ↑ mitochondrial activity	Thornton et al. (2016)
		↑ FAP proliferation and ↓ adipogenic differentiation	Kang et al. (2018)
		Biphasic dose-dependent mitochondrial respiration effects	Nadeau et al. (2019)
	Adipose tissue	↓ Adipocyte differentiation and WAT mass	Fuster et al. (2011)
		Muscle IL-15Rα deletion → ↓ IL-15 secretion, ↓ anti-obesity protection	Zumbaugh et al. (2021)
BDNF	Muscle	AMPK → ↑ FA oxidation	Matthews et al. 2009)
		AMPK–PINK1–Parkin → mitophagy	Ahuja et al. (2022)
		AMPK–DRP1–MFF → mitochondrial fission	Ahuja et al. (2022)
		PPARδ-dependent lipid utilization and post-exercise recovery	Chan et al. (2024)
		Fiber-type specification → glycolytic shift with overexpression	Delezie et al. (2019)
	Pancreas	TrkB.T1 isoform in β-cells → insulin secretion; muscle-specific BDNF KO → impaired glucose tolerance resembling β-cell TrkB.T1 deletion	Fulgenzi et al. (2020)
METRNL	Muscle	Stat3/IGF-1 → ↑ satellite cell proliferation and macrophage transition to anti-inflammatory phenotype	Baht et al. (2020)
		AMPK- or PPARδ-dependent ↑ insulin sensitivity	Jung et al. (2018a)
		TNFα-driven apoptosis of FAPs → ↓ fibrosis in aged/injured muscle	Lee et al. (2022a)
	Adipose tissue	Eosinophil–IL-4/IL-13 axis → alternative macrophage activation → ↑ beige-fat thermogenesis and mitochondrial oxidation	Rao et al. (2014)
		PPARγ → ↑ insulin sensitivity; ↓ adipogenesis in SVF context	Javaid et al. (2021); Löffler et al. (2017)
	Heart	AMPK/SIRT1 → ↓ hypertrophy and dysfunction	Cao et al. (2024)
		↓ Doxorubicin-induced cardiotoxicity	Hu et al. (2020)
		KIT receptor-dependent angiogenesis in infarct repair	Reboll et al. (2022)
SPARC	Muscle	Direct actin binding in myofibers → ↑ force recovery post-fatigue	Jørgensen et al. (2017)
		AMPK activation (Ca^2^⁺-dependent) → ↑ glucose tolerance	Aoi et al. (2019)
		IGF-I → PI3K → ↑ SPARC → ↓ intramuscular fat accumulation	Mathes et al. (2022)
		↑ Mitochondrial function, ↑ GLUT and OXPHOS proteins in aging	Ghanemi et al. (2022)
	Adipose tissue	Wnt/β-catenin → ↓ adipogenesis	Nie and Sage (2009)
		FGF2/miR-29a/SPARC axis → ↑ intramuscular adipogenesis in aging	Mathes et al. (2021)
		↑ NLRP3 inflammasome in inflammatory contexts	Ryu et al. (2023)
FGF21	Muscle	PI3K/Akt/mTOR → induction by electrical stimulation	Arias-Calderón et al. (2023)
		PI3K/PKC-ζ → GLUT4 → ↑ glucose uptake	Rosales-Soto et al. (2020)
		mTOR–YY1–PGC1α → mitochondrial adaptation	Ji et al. (2015)
		Bnip3-mediated mitophagy in fasting/atrophy	Oost et al. (2019)
		TGF-β1/p38 MAPK → fiber-type conversion	Luo et al. (2023)
	Adipose tissue	β-Klotho/FGFR1c → ↑ lipolysis and WAT browning	Itoh (2014); Fisher and Maratos-Flier (2016)
	Liver	↑ FA oxidation; AMPK-dependent lipophagy → ↓ MASLD	Gao et al. (2020)
	Heart	AMPK/FOXO3/SIRT3 → cardioprotection; ↓ fibrosis in diabetic cardiomyopathy and MI	Jin et al. (2022); Ma et al. (2021)
	Vasculature	↓ Pyroptosis in exercise settings	Li et al. (2022)
BAIBA	Muscle	IRS-1/Akt and AMPK–PPARδ → ↑ mitochondrial FA oxidation	Roberts et al. (2014)
		↑ Insulin signaling; ↓ NF-κB-driven inflammation	Jung et al. (2015)
	Adipose tissue	↑ UCP1, ↑ thermogenic genes → ↑ browning	Roberts et al. (2014)
		↑ FA oxidation; AMPK/IRS-1 → ↑ insulin sensitivity; ↓ NF-κB	Jung et al. (2018b)
	Liver	AMPK → ↑ β-oxidation	Roberts et al. (2014)
		↓ Endoplasmic reticulum stress and hepatic apoptosis	Shi et al. (2016)
	Bone	MRGPRD signaling → ↓ oxidative stress-induced osteocyte death	Kitase et al. (2018)
		Wnt/TGFβ/BMP → ↑ bone formation (L-BAIBA)	Prideaux et al. (2023)
		Enantiomer-specific associations with BMD and functional performance	Lyssikatos et al. (2023)
LIF	Muscle	JunB/c-Myc → ↑ myoblast proliferation	Broholm et al. (2012)
		JAK2–STAT3 → ↑ satellite cell proliferation	Spangenburg and Booth (2002)
		PI3K/mTORC2/Akt → ↑ glucose uptake (AMPK-independent)	Brandt et al. (2015)
		In dystrophic muscle → ↓ Th2 cytokines, ↓ pro-fibrotic macrophage phenotype, ↓ FAP activity	Welc et al. (2019)
	Bone	LIFRβ–gp130 → STAT3 → ↑ osteoblast differentiation and proliferation (local cytokine action)	Sims and Johnson (2012)
Apelin	Muscle	APJ (GPCR) → AMPK and Akt → ↑ glucose uptake, ↑ FA oxidation, ↑ mitochondrial oxidative capacity	Attané et al. (2012); Yue et al. (2010)
		IGF-1 signaling → fiber-type remodeling	Kilpiö et al. (2024)
		Tead1 regulates apelin secretion in myogenic cells	Lee et al. (2022b)
		APJ enriched in endothelial cells → paracrine crosstalk for muscle repair	Lee et al. (2022b)
		Apelin–APJ attenuates CKD-induced muscle wasting	Enoki et al. (2023)
	Adipose tissue	APJ → thermogenesis and metabolic regulation	Than et al. (2015)
		Maternal exercise → offspring metabolic health via apelin	Son et al. (2022)
	Gut	APJ–AMPK → ↑ mitochondrial biogenesis and FA metabolism in duodenal epithelium	Chae et al. (2023)
Musclin	Muscle	NPR-C/NPR3 binding → ↑ ANP/cGMP–PGC1α → ↑ mitochondrial biogenesis and endurance	Moffatt et al. (2007); Subbotina et al. (2015)
		FILIP1L → ↑ macrophage phagocytosis of apoptotic FAPs → ↓ fibrosis	Kang et al. (2024)
		PGC1α-dependent → ↓ cancer cachexia-induced wasting	Re Cecconi et al. (2019)
		Insulin-resistant context: recombinant musclin ↓ insulin-stimulated glucose uptake and glycogen synthesis	Nishizawa et al. (2004)
	Adipose tissue	In vitro PKA/p38 → ↑ lipolysis, ↓ lipogenesis	Choi et al. (2023)
		In vivo Tfr1 binding → ↓ cAMP/PKA → ↓ beige-fat thermogenesis (temperature-sensitive)	Jin et al. (2023)
	Liver	↑ SIRT7 → ↑ autophagy → ↓ lipid accumulation and ER stress	Cho et al. (2023)

*AMPK AMP*-activated protein kinase, *ActRII* activin receptor type II, *ALK* activin receptor-like kinase, *ALCAT1* acyl-CoA:lysocardiolipin acyltransferase 1, *ANP* atrial natriuretic peptide, *BAIBA* β-aminoisobutyric acid, *BDNF* brain-derived neurotrophic factor, *BMP* bone morphogenetic protein, *cAMP* cyclic adenosine monophosphate, *cGAS* cyclic GMP-AMP synthase, *cGMP* cyclic guanosine monophosphate, *CKD* chronic kidney disease, *DRP1* dynamin-related protein 1, *ER* endoplasmic reticulum, *ERK* extracellular signal-regulated kinase, *FA* fatty acid, *FAP* fibro-adipogenic progenitor, *FGF21* fibroblast growth factor 21, *FGFR* fibroblast growth factor receptor, *FNDC5* fibronectin type III domain-containing protein 5, *FOXO* forkhead box O, *FSTL-3* follistatin-like 3, *GASP-1* growth and differentiation factor–associated serum protein 1, *GDF11* growth differentiation factor 11, *GLP-1* glucagon-like peptide-1, *GLUT* glucose transporter, *gp130* glycoprotein 130, *GSK3* glycogen synthase kinase 3, *HSL* hormone-sensitive lipase, *IGF-1* insulin-like growth factor 1, *IL* interleukin, *IRS-1* insulin receptor substrate 1, *JAK* Janus kinase, *KLF15* Krüppel-like factor 15, *LIF* leukemia inhibitory factor, *LIFRβ* leukemia inhibitory factor receptor β, *MAPK* mitogen-activated protein kinase, *METRNL* meteorin-like, *MFF* mitochondrial fission factor, *MI* myocardial infarction, *MRGPRD* MAS-related G-protein coupled receptor D, *mtDNA* mitochondrial DNA, *MuRF-1* muscle RING-finger protein-1, *NPR* natriuretic peptide receptor, *Nrf2* nuclear factor erythroid 2–related factor 2, *OXPHOS* oxidative phosphorylation, *PGC1α* peroxisome proliferator-activated receptor γ coactivator 1-alpha, *PI3K* phosphoinositide 3-kinase, *PINK1* PTEN-induced kinase 1, *PKA* protein kinase A, *PPAR* peroxisome proliferator-activated receptor, *RANKL* receptor activator of nuclear factor κB ligand, *REV-ERBα* reverse Erb-α, *SIRT* sirtuin, Smad mothers against decapentaplegic homolog, *SPARC* secreted protein acidic and rich in cysteine, *SREBP2* sterol regulatory element-binding protein 2, *STAT* signal transducer and activator of transcription, *STING* stimulator of interferon genes, *TGF-β* transforming growth factor-β, *Tfr1* transferrin receptor 1, *TrkB* tropomyosin receptor kinase B, *UCP1* uncoupling protein 1, *WAT* white adipose tissue

**Table 2:** Evidence-based assessment of the translational potential of myokines

Myokine (Tier)	Evidence level	Source certainty	Receptor/target status	Key causal evidence	Human evidence	Translational potential
IL-6 (Tier 1)	Cell Animal Human	Moderate: exercise-induced release from contracting muscle is well supported, but also secreted by adipose tissue and immune cells	Well-defined: IL-6Rα/gp130; classical vs. trans-signaling distinction established	RCT: IL-6R blockade (tocilizumab) abolished exercise-induced visceral fat loss; IL-6 KO mice: blunted exercise adaptation	Acute response up to 100-fold in plasma; RCT interventional data available	High: causal biology established; olamkicept in clinical development for inflammatory disease
Irisin/FNDC5 (Tier 2)	Cell Animal Human (limited)	Moderate: increases in muscle with acute aerobic training, yet antibody/ELISA specificity and cross-reactivity remain major confounders; also expressed in adipose and other organs	Partially defined: integrin αVβ5 identified in bone, heart, kidney; incomplete across metabolic tissues	Recombinant irisin rescues sarcopenic phenotypes in aged mice; muscle-specific FNDC5 KO studies	Acute exercise-induced increase confirmed by mass spectrometry; chronic training effects heterogeneous across meta-analyses	Moderate: biologics engineering strategies under investigation; limited by source ambiguity and absent human mechanistic interventions
Myostatin (Tier 1)	Cell Animal Human	High: canonical muscle-enriched negative regulator	Well-defined: ActRIIB/ALK4/5 → Smad2/3; non-Smad pathways also characterized; ActRII-directed interventions also affect activins and GDF11	KO/neutralizing Ab/ligand traps; multiple human trials (myostatin Ab, ActRII blockade)	Pharmacological trials (bimagrumab, LY2495655) with lean mass and limited functional outcomes; genetic and intervention data	High: most clinically advanced myokine pathway; still unresolved “mass → function” translation
GDF11 (Tier 3)	Cell Animal (conflicting) Human (limited)	Low: multiple tissue sources; cross-reactivity with myostatin in immunoassays complicates source attribution	Plausible ActRII-like/Smad2/3; not independently validated; confounded by myostatin overlap in most inhibitor studies	Conflicting animal data: recombinant GDF11 “rejuvenation” vs inhibitory/wasting phenotypes	Observational only; some association with healthy aging phenotypes in lifelong exercisers; no mechanistic human interventions	Low: biologically unresolved; not a viable independent therapeutic target pending assay and mechanistic resolution
IL-15 (Tier 2)	Cell Animal Human (limited)	Moderate: muscle expresses IL‑15, but immune cells also contribute; circulating IL-15 not muscle-specific	Relatively well-defined: IL-15Rα/IL-2Rβ/γc complex	Muscle-specific IL-15Rα KO impairs protection against obesity; IL-15 TG → improved muscle quality	Meta-analysis supports acute exercise-induced increase; chronic effects heterogeneous; no causal human intervention data	Moderate: credible receptor axis and preclinical metabolic effects; limited by immune pleiotropy and non-muscle source contributions
BDNF (Tier 2)	Cell Animal Human (limited)	Low: brain and platelets are major contributors to circulating BDNF; muscle predominantly expresses pro-BDNF	Well-defined: TrkB (NTRK2)	Muscle-specific BDNF KO → metabolic dysfunction, obesity	Exercise-induced increase confirmed in meta-analyses of RCTs; source attribution unresolved	Moderate: receptor axis well-defined but endocrine myokine role unresolved; best viewed as local autocrine/paracrine mediator
METRNL (Tier 2)	Cell Animal Human (limited)	Moderate: induced in muscle after exercise and in adipose with cold, but immune cell origin is prominent in injury/regeneration contexts	Partially defined: KIT receptor tyrosine kinase identified in cardiac endothelial repair; relevance to skeletal muscle and adipose metabolism unclear	Whole-body and macrophage-specific METRNL KO → impaired muscle regeneration	Acute exercise increases circulating METRNL; 12-week RCT in older adults shows correlations with inflammatory markers; no mechanistic human interventions	Moderate: biologically plausible; human evidence incomplete; receptor generalizability across metabolic tissues unresolved
SPARC (Tier 3)	Cell Animal Human (limited)	Moderate: muscle is major exercise-responsive source; circulating response is protocol-dependent and not uniformly observed	Not fully defined: AMPK activation via Ca^2^⁺/voltage-dependent Ca^2^⁺ channel interaction; no canonical receptor established	SPARC-deficient mice → impaired post-fatigue recovery; recombinant SPARC → AMPK-dependent glucose uptake rescue	Acute aerobic exercise increases circulating SPARC in humans; no mechanistic human intervention studies or evidence of causal metabolic effect	Low: recombinant protein proof-of-concept in mice; absent receptor definition and human mechanistic evidence limit translation
FGF21 (Tier 1)	Cell Animal Human	Moderate: liver is dominant endocrine source; muscle contribution is context/stress-dependent	Well-defined: β-Klotho coreceptor + FGFR1c (primary)/FGFR3c	FGF21 analogue RCT (LY2405319): improved metabolism in obese T2DM adults; pegozafermin Phase 2b: histologic improvement in MASH	Strong pharmacological evidence; circulating FGF21 direction varies by exercise modality in meta-analyses; elevated in obesity/T2DM as compensatory response	High: most mature receptor system; FGF21-based drugs in late-stage development; therapeutic framing is pharmacological rather than exercise-mimetic
BAIBA (Tier 3)	Cell Animal Human (limited)	Moderate: released from myocytes in PGC‑1α/exercise biology; enantiomer sources differ and tissue-of-origin in humans unclear	Partially defined: MRGPRD identified as high-affinity target for L-BAIBA in osteocyte biology; not established in other metabolic tissues	Supplementation in vivo; receptor antagonism/KO strategies in osteocytes support mechanism in bone	Exercise increases both enantiomers in humans; genetic (AGXT2) influence confirmed; no causal intervention studies	Low–moderate: promising preclinical myometabolite; receptor mapping incomplete outside bone; human evidence associative
LIF (Tier 3)	Cell Animal Human (limited)	Moderate: contraction induces muscle LIF expression; plasma LIF may be undetectable in humans, supporting mainly paracrine/autocrine action	Well-defined: LIFRβ–gp130	LIFR knockdown abolishes while recombinant LIF induces myoblast proliferation	mRNA induction in muscle after endurance and resistance exercise; LIF undetectable in plasma post-exercise; no circulating endocrine evidence in humans	Low: autocrine/paracrine mode limits systemic therapeutic use; local delivery approaches may be more feasible
Apelin (Tier 1)	Cell Animal Human	Moderate: both muscle and adipose tissue express and secrete apelin; circulating levels reflect mixed tissue contributions	Well-defined: APJ (APLNR), a GPCR	Apelin or APLNR deficiency accelerates dysfunction with aging; apelin supplementation restores function	Acute exercise-induced increases in humans; APJ infusion → cardiovascular effects in humans; first-in-human APJ agonist trial completed	High-moderate: strong preclinical causal support; cardiovascular safety profile and exercise-specific causal contribution need further definition
Musclin (Tier 3)	Cell Animal Human (limited)	Moderate: enriched in skeletal muscle; obesity-associated expression changes; systemic levels influenced by metabolic state	Partially defined: NPR-C/NPR3 (natriuretic peptide clearance receptor); Tfr1 in beige adipocytes	Musclin KO mice decreases exercise tolerance; recombinant musclin rescues endurance phenotype	Primary myoblast secretion data; obesity-associated circulating changes; no robust human trials	Low–moderate: biologically plausible with strong preclinical support; sparse human causal evidence limit immediate translation

Evidence level indicators (Cell/Animal/Human) denote the experimental systems in which supportive data have been generated; qualifiers in parentheses (“limited”, “conflicting”) reflect the robustness of the available evidence. Tier classifications reflect human causal evidence and receptor definition, weighted in that order of priority: Tier 1, pathways with human interventional or causal evidence; Tier 2, receptor-defined preclinical mediators with consistent but largely correlative human signals; Tier 3, exploratory or hypothesis-generating candidates whose mediator status, receptor identity, or assay validity remains insufficiently resolved. Translational potential classifications reflect the authors’ qualitative integrative assessment based on the framework of receptor definition, exposure control, and safety discussed in Section IV

*Ab* antibody, *AMI* acute myocardial infarction, *APJ/APLNR* apelin receptor, *BMD* bone mineral density, *CKD* chronic kidney disease, *ELISA* enzyme-linked immunosorbent assay, *FGF21* fibroblast growth factor 21, *FGFR* fibroblast growth factor receptor, *GPCR* G protein-coupled receptor, *IL* interleukin, *KO* knockout, *METRNL* meteorin-like, *MI* myocardial infarction, *MRGPRD MAS*-related G-protein coupled receptor D, *MSTN* myostatin, *MASH* metabolic dysfunction-associated steatohepatitis, *NPR-C/NPR3* natriuretic peptide receptor C, *RCT* randomized controlled trial, *T2DM* type 2 diabetes mellitus, *Tfr1* transferrin receptor 1, *TG* transgenic, *TrkB* tropomyosin receptor kinase B, *SPARC* secreted protein acidic and rich in cysteine

**Table 3:** Natural and synthetic compounds regulating myokine expression and activity

Myokine	Modulating compound	Compound category	Regulatory effect	Regulatory mechanism	Experimental model	References
IL-6	Ouabain	Natural product	↑ IL-6 secretion; ↓ IL-6 signaling	↓ STAT3 total protein and phosphorylation (via proteasome-mediated degradation); ↑ ERK1/2 and Akt-mTOR pathway activation; no effect in ouabain-resistant NKA-expressing cells	Human skeletal muscle cells (in vitro)	Pirkmajer et al. (2020)
	Caffeine	Natural product	↑ IL-6 secretion from muscle	↑ IL-6 in skeletal muscle via p38/MAPK activation; induces liver STAT3 signaling for MASLD protection	Myotubes (in vitro); mice (in vivo)	Fang et al. (2019)
	Bazedoxifene	Approved drug	↓ IL-6 levels; ↓ STAT3 phosphorylation	Inhibits IL-6/IL-6R/STAT3 pathway; suppresses VSMC proliferation and stabilizes endothelium via blocking sIL-6R-mediated trans-signaling	Endothelial cells/VSMCs (in vitro); ApoE-deficient mice (in vivo)	Luo et al. (2021)
Irisin/FNDC5	Icariin	Natural product	↑ FNDC5/irisin expression	AMPK → PGC-1α → FNDC5 induction	C2C12 cells (in vitro); mice (in vivo)	Chen et al. (2019)
	Sesamin	Natural product	↑ FNDC5/irisin expression and secretion	SIRT1/SMAD3 axis → ↑ PGC-1α; ↓ p-SMAD3	Mouse skeletal muscle/mice (in vivo)	Kou et al. (2022)
	Ursolic acid	Natural product	↑ FNDC5 expression and plasma irisin	Enhances FNDC5/irisin axis with aerobic training; ↑ UCP1	Rats (in vivo)	Teimourian et al. (2020)
	Pentamethylquercetin	Natural product	↑ FNDC5/irisin expression and secretion	AMPK → PGC-1α → FNDC5; promotes browning via muscle–adipose crosstalk	Murine myocytes (in vitro); obese mice (in vivo)	Wu et al. (2022a)
	Caffeine	Natural product	↑ FNDC5/irisin expression and secretion	Calcium-dependent signaling → ↑ PGC-1α	Myotubes (in vitro); HFD-fed mice (in vivo)	Liu et al. (2023)
	Quercetin	Natural product	↑ FNDC5 and PGC-1α expression in skeletal muscle; ↑ plasma irisin	Activates AMPK → ↑ PGC-1α → ↑ FNDC5 transcription; *Pgc-1α* silencing abolishes effect	L6 myotubes, palmitate-treated (in vitro); HFD-fed rats (in vivo)	Muscia Saez et al. (2026)
	Resveratrol + all-trans retinoic acid	Small molecule	↑ FNDC5 gene expression (combination only)	Synergistic induction of FNDC5 mRNA	C2C12 myotubes (in vitro)	Abedi-Taleb et al. (2019)
	Liraglutide	Approved drug	↑ FNDC5 expression and irisin secretion	↑ PGC1α and AMPK; promotes WAT browning via irisin-mediated AMPKα/ACC1/UCP1 signaling	C2C12 cells (in vitro); mice (in vivo)	Zhang et al. (2024)
	FGF19	Peptide	↑ FNDC5/irisin expression	AMPK/SIRT1/PGC-1α activation	Palmitic acid-treated C2C12 myotubes (in vitro); HFD-fed mice (in vivo)	Guo et al. (2021)
Myostatin	Licochalcone A and B	Natural product	↓ MSTN expression/serum levels	Direct binding to MSTN inhibits MSTN–ActRIIB interaction; ↓ SMAD2/3, Atrogin1, and MuRF1	C2C12 myoblasts (in vitro); mice (in vivo)	Ahmad et al. (2024)
	Cedrol derivative	Natural product	↓ MSTN and MuRF1 expression	MOR23 → Ca^2 +^ signaling → phospho-CaMKII/FoxO3a-mediated repression of MSTN transcription	Mice (in vivo)	Kang et al. (2025)
	EGCG	Natural product	↓ MSTN expression; ↓ MuRF1/Atrogin-1	↑ miR-486-5p → ↑ AKT phosphorylation → ↓ FoxO1a activity	Late-passage C2C12 myoblasts *(*in vitro); aged mice (in vivo)	Chang et al. 2020)
	Curcumin	Natural product	↓ MSTN expression in skeletal muscle	Suppresses NF-κB and UPS; improves mitochondrial function and ↑ myogenin	Mice (in vivo)	Zhang et al. (2022)
	Glycyrrhiza uralensis extract	Natural product	↓ MSTN expression in DEX-induced sarcopenia	↓ TNF-α, MuRF1, and MAFbx via FoxO3a phosphorylation	C2C12 myoblasts (in vitro); dexamethasone-treated mice (in vivo)	Zheng et al. (2024)
	IMB0901	Small molecule	↓ MSTN expression and activity; ↓ Atrogin-1 and MuRF-1	Inhibits MSTN promoter/signaling pathway; suppresses UPS; enhances AKT/mTOR-mediated protein synthesis	C26 cancer cachexia model mice (in vivo)	Liu et al. (2019a)
IL-15	Eugenol	Natural product	↑ IL-15 expression in skeletal muscle	TRPV1 → CaN/NFATc1 signaling	C2C12 myotubes (in vitro); mice (in vivo)	Huang et al. (2024)
	Hydroxylated vitamin D3 metabolites	Small molecule	↑ IL-15 expression in skeletal muscle	VDR-dependent regulation of IL-15	C2C12 myotubes (in vitro); Vdr-KO mice and rats (in vivo)	Ewendt et al. (2025)
	Benzoic acid-derived IL-15Rα antagonists	Small molecule	↓ IL-15 signaling; ↓ PBMC proliferation, TNF-α, and IL-17	IL-15Rα binding inhibition	PBMCs (ex vivo*/*in vitro)	Krzeczynski et al. (2023)
BDNF	Oleuropein	Natural product	↑ BDNF expression and neuroprotection	CREB-mediated BDNF transcription; ↑ Akt and GSK-3β phosphorylation	Rotenone-induced Parkinson’s disease mice (in vivo)	Singh et al. (2023)
	Gastrodin	Natural product	↑ BDNF expression and nerve regeneration	Inhibits miR-497, a negative regulator of BDNF	Rat peripheral nerve injury model (in vivo)	Yongguang et al. (2022)
	Phloridzin	Natural product	↑ BDNF expression in brain	Mitigates oxidative stress; ↑ BDNF, TrkB, CREB, and ERK signaling	Type 2 diabetic mice (in vivo)	Kamdi et al. (2021)
	Lactate	Small molecule	↑ Hippocampal BDNF	SIRT1 → PGC-1α → FNDC5 → BDNF/TRKB signaling	Mice (in vivo)	El Hayek et al. (2019)
SPARC	Madecassoside	Natural product	↑ SPARC expression; ↑ angiogenesis/cardioprotection	PKC-β-dependent SPARC upregulation	Endothelial cells (in vitro); rat myocardial infarction model (in vivo)	Wang et al. (2025)
	(−)-Epicatechin	Natural product	↑ SPARC expression	↑ BMP2/SPARC/RUNX2 osteogenic program	Human bone marrow-derived mesenchymal stem cells (in vitro)	Palma-Lara et al. (2025)
FGF21	Baicalein	Natural product	↑ FGF21 mRNA and protein secretion	RORα → CHOP → FGF21 induction	C2C12 myotubes (in vitro)	Hirai et al. (2019b)
	Sulforaphane	Natural product	↑ FGF21 and FGFR1 expression	Upregulates FGF21/FGFR1 axis; ↓ p38 MAPK phosphorylation	FFA-treated HepG2 cells (in vitro); MASLD mice (in vivo)	Wu et al. (2022b)
	Berberine	Natural product	↑ FGF21 expression in BAT	AMPK → Bmal1 → FGF21 transcription	Brown adipocytes (in vitro); mice (in vivo)	Hirai et al. (2019a)
	Maresin 1	Small molecule	↓ Hepatic FGF21 expression; ↑ FGF21 pathway responsiveness	Inhibits hepatic PPARα; ↑ β-Klotho/FGFR1/EGR1/cFOS in liver and WAT	Mice (in vivo)	Martínez-Fernández et al. (2019)
	Dimethyl itaconate	Small molecule	↑ FGF21 expression	AMPK → FGF21 → PPARδ signaling	Palmitate-treated C2C12 myocytes (in vitro)	Park et al. (2021)
	SR1078	Small molecule	↑ FGF21 mRNA and protein secretion	RORα → CHOP → FGF21 induction	C2C12 myotubes (in vitro)	Hirai et al. (2019b)
	Liraglutide	Approved drug	↑ FGF21 secretion in WAT and macrophages	FGF21 → FGFR3 → LKB1–AMPK–ACC1 signaling	Macrophages (in vitro); type 2 diabetic mice (in vivo)	Zhang et al. (2021)
	Sacubitril/valsartan	Approved drug	↑ Serum FGF21; cardioprotection	PPAR agonism and FGF21-dependent cardioprotection	AMI patients (clinical); MI mice (in vivo)	Wei et al. (2025)
	Empagliflozin	Approved drug	↑ hepatic FGF21 mRNA and plasma FGF21 levels	↑AMPKα/ACC phosphorylation in skeletal muscle; lipolysis in adipose tissue	HFD-induced obese C57BL/6J mice (in vivo)	Xu et al. (2017)
Apelin	(−)-Epicatechin	Natural product	↑ Apelin and APJ receptor expression in adipose tissue	↑ AMPKα phosphorylation; modulates lipolysis, lipogenesis, and adipogenesis	Rats (in vivo)	De Los Santos et al. (2023)
	Pyrazole-based agonist	Small molecule	Mimics apelin receptor activation	Apelin receptor agonism improves body weight, glucose utilization, and liver steatosis	Diet-induced obesity mice (in vivo)	Narayanan et al. (2021)
	17β-Estradiol	Small molecule	↑ Apelin expression in right ventricle	ER-α → BMPR2-dependent induction of apelin	Pulmonary hypertension rodent models (in vivo)	Frump et al. (2021)

*AKT* protein kinase B, *AMPK AMP*-activated protein kinase, *ApoE* apolipoprotein E, *BAT* brown adipose tissue, *BDNF* brain-derived neurotrophic factor, Bmal1 brain and muscle Arnt-like 1, *BMP2* bone morphogenetic protein 2, *BMPR2* bone morphogenetic protein receptor 2, *CaMKII* Ca^2^⁺/calmodulin-dependent protein kinase II, *CaN* calcineurin, *CHOP C/EBP* homologous protein, *CREB cAMP* response element-binding protein, *EGCG* epigallocatechin-3-gallate, *ER-α* estrogen receptor α, *ERK* extracellular signal-regulated kinase, *FFA* free fatty acid, *FGF21* fibroblast growth factor 21, *FGFR1* fibroblast growth factor receptor 1, *FNDC5* fibronectin type III domain-containing protein 5, *FoxO* forkhead box O, *GLP-1* glucagon-like peptide-1, *GSK-3β* glycogen synthase kinase-3β, *HFD* high-fat diet, *IL* interleukin, *IL-6R* interleukin-6 receptor, *LKB1* liver kinase B1, *MAFbx* muscle atrophy F-box, *MAPK* mitogen-activated protein kinase, *MI* myocardial infarction, *MSTN* myostatin, *MuRF1* muscle RING-finger protein 1, *MASLD* metabolic dysfunction-associated steatotic liver disease, *NF-κB* nuclear factor kappa-light-chain-enhancer of activated B cells, *NFATc1* nuclear factor of activated T cells c1, *PBMC* peripheral blood mononuclear cell, *PGC-1α* peroxisome proliferator-activated receptor γ coactivator 1-alpha, *PKC-β* protein kinase C β, *PPAR* peroxisome proliferator-activated receptor, *RORα* retinoic acid receptor-related orphan receptor α, *RUNX2* runt-related transcription factor 2, *SGLT-2* sodium-glucose cotransporter 2, *SIRT1* sirtuin 1, *sIL-6R* soluble interleukin-6 receptor, *SMAD* mothers against decapentaplegic homolog, *SPARC* secreted protein acidic and rich in cysteine, *STAT*3 signal transducer and activator of transcription 3, *TNF-α* tumor necrosis factor-α, *TRPV*1 transient receptor potential vanilloid 1, *UCP1* uncoupling protein 1, *UPS* ubiquitin–proteasome system, *VDR* vitamin D receptor, *VSMC* vascular smooth muscle cell, *WAT* white adipose tissue

## Myokine biology and evidence for metabolic action

### Interleukin-6

IL−6 is a prototypical myokine released from skeletal muscles during physical activity, although circulating IL-6 is not derived exclusively from muscle, as adipose tissue and immune cells can also contribute to its systemic levels (Lyngso et al. 2002; Han et al. 2020). In humans, exercise elevates circulating IL-6 levels by up to 100-fold in proportion to its duration and intensity (Pedersen and Febbraio 2008), with a recent combined human, mouse, and cell study showing that high-intensity exercise triggers protease- and lactate-dependent IL-6 release from intramyocellular vesicles, linking secretion to energy demand (Hojman et al. 2019). Mechanistically, IL-6 signals through the IL-6 receptor-α (IL-6Rα) and gp130 receptor complex, with membrane-bound IL-6Rα mediating classical signaling and soluble IL-6R enabling trans-signaling in gp130-expressing cells (Garbers et al. 2011; Reeh et al. 2019). This dual signaling architecture underlies a defining feature of IL-6 biology—its context dependence. During exercise, IL-6 is generally linked to transient metabolic adaptation and anti-inflammatory responses, whereas in chronic disease states sustained IL-6 exposure, particularly via trans-signaling, is more often associated with inflammatory pathology (Nash et al. 2023). The biological outcome is further shaped by exposure duration, tissue context, receptor availability, and the surrounding cytokine milieu (Rose-John et al. 2023).

In skeletal muscle, animal and cell studies indicate that an acute increase in IL-6 promotes satellite cell–mediated hypertrophy (Serrano et al. 2008), enhances glycogen breakdown and lipolysis via AMPK (Kelly et al. 2009), and increases GLUT4 expression and insulin sensitivity (Ikeda et al. 2016). IL-6 also shows fiber-type–specific expression, with higher expression in oxidative fibers than in glycolytic fibers in response to stimulation (Liang et al. 2018), and contributes to exercise-induced mitochondrial remodeling and autophagy via REV–ERBα, with these responses impaired in the absence of IL-6 signaling (Pinto et al. 2022). These beneficial acute effects contrast with the consequences of chronic IL-6 elevation, which can impair mitochondrial function and increases fatigue through gp130-dependent signaling, even without major changes in muscle fiber type or mass (Vanderveen et al. 2019). Consistent with this catabolic pattern, in immobilized mice, downregulation of myofiber-specific Piezo1 activates the KLF15–IL-6 axis to promote atrophy, a response absent in IL-6 knockout mice (Hirata et al. 2022).

Substantial evidence supports a role for IL-6 in interorgan metabolic crosstalk. In adipose tissue, animal studies and human intervention data support the concept that IL-6 acts as an “energy allocator”, stimulating lipolysis and fat oxidation during exercise (Wedell-Neergaard et al. 2019) and promoting browning via UCP1 expression (Knudsen et al. 2014). Notably, a randomized controlled trial in humans demonstrated that IL-6 receptor blockade with tocilizumab abolished exercise-induced visceral fat loss, providing strong causal evidence that IL-6 signaling contributes to the metabolic benefits of training (Wedell-Neergaard et al. 2019). In parallel, mouse studies showed that IL-6 deficiency blunted exercise-associated reductions in adiposity and hepatic steatosis (Li et al. 2021), and female inducible muscle-specific IL-6 knockout mice displayed impaired basal lipolysis, exercise-driven glyceroneogenesis, and anti-inflammatory responses in visceral and subcutaneous fat (Bertholdt et al. 2020).

Evidence for IL-6 action extends further to postprandial metabolism and muscle-bone crosstalk. In a randomized controlled human study, exogenous IL-6 delayed gastric emptying and lowered postprandial glycemia independently of glucagon-like peptide-1 (GLP-1), while reducing insulin secretion via GLP-1–dependent pathways in humans (Lang Lehrskov et al. 2018). In addition, a mouse genetic study demonstrated that muscle-derived IL-6 promotes osteocalcin release from osteoblasts, thereby establishing a muscle–bone axis that enhances nutrient uptake in myofibers and supports exercise capacity (Chowdhury et al. 2020).

Overall, IL-6 is among the myokines with the most robust human causal evidence, supported by tocilizumab intervention data together with consistent human, animal, and cell-based findings. Its defining feature, however, is context dependence. Acute contraction-associated IL-6 appears metabolically beneficial whereas chronic elevation may contribute to muscle dysfunction, fatigue, and inflammatory remodeling. However, several proposed muscle-autonomous effects still rely mainly on cell and animal models, and muscle-to-adipose crosstalk remains inferred rather than directly demonstrated in humans.

### Irisin/FNDC5

Fibronectin type III domain-containing protein 5 (FNDC5), proteolytically cleaved to generate irisin, has emerged as an exercise-responsive myokine involved in metabolic adaptation and interorgan communication. The original mouse and human study identifying FNDC5/irisin as a PGC1α-dependent factor linked to exercise and adipose tissue browning prompted substantial interest in irisin as a mediator of the systemic benefits of physical activity (Boström et al. 2012). Experimental studies in rodents and cultured muscle cells further showed that FNDC5/irisin can be induced by exercise-related pathways, including IL-6/STAT3 and PGC1α signaling (Arabzadeh et al. 2022; Nguyen et al. 2024). However, FNDC5 is also expressed in adipose tissue and other organs, so circulating irisin in humans cannot be attributed exclusively to skeletal muscle (Moreno-Navarrete et al. 2013).

Within skeletal muscle, cell and animal studies support multiple roles for irisin spanning metabolism, myogenesis, and protection against atrophy. Metabolically, irisin promotes muscle glucose and lipid metabolism via AMPK activation (Huh et al. 2014; Lee et al. 2015). In myogenic contexts, FNDC5/irisin is enriched in slow-twitch fibers and early myogenesis, participating in a positive regulatory loop involving PGC1α during myogenic differentiation (Lavi et al. 2022), and promotes muscle growth through IL-6-mediated myoblast differentiation, fusion, and hypertrophy (Reza et al. 2017). In the context of muscle loss, skeletal muscle FNDC5/irisin expression and circulating irisin decline with age, while recombinant irisin improves sarcopenic phenotypes by suppressing MuRF-1 and Atrogin-1 and improving mitochondrial function (Guo et al. 2023). Similar protective effects extend to other catabolic settings: irisin attenuates dexamethasone-induced atrophy through IGF-1/Akt/ERK activation and FoxO3α inhibition (Chang and Kong 2020), and reduces fibrosis and oxidative stress in D-galactose models via the PI3K/Akt–Nrf2 and NOX4/TGF-β1/Smad signaling (Wu et al. 2023). It also protects against chronic kidney disease (CKD)- and myocardial infarction–induced muscle wasting by modulating FOXO3 and ALCAT1 signaling (Ren et al. 2022; Wang et al. 2024).

Beyond skeletal muscle, irisin participates in multi-organ crosstalk, with a mechanistic advance provided by the identification of integrin αVβ5 as an irisin receptor. In bone, mouse studies show that irisin modulates bone homeostasis, with evidence for both enhanced bone formation (Colaianni et al. 2015) and increased bone resorption (Kim et al. 2018). Sex-specific effects have also been reported: male but not female FNDC5-knockout mice show increased bone mass but reduced bone quality, with alterations in the osteocyte transcriptome (Shimonty et al. 2024). Consistent with the receptor framework, exercise-induced irisin enhances osteoblastogenesis in male mice (Hatakeyama et al. 2025). The irisin-integrin axis further promotes osteogenesis via the ERK/STAT and BMP/Smad pathways (Xue et al. 2022), activates Akt to counter diabetic cardiomyopathy in the heart (Lin et al. 2021), and blocks mtDNA leakage and cGAS–STING inflammation in acute kidney injury (Peng et al. 2025). In adipose tissue, irisin induces browning of subcutaneous fat via p38 MAPK and ERK1/2 signaling (Zhang et al. 2014), whereas FNDC5 overexpression enhances lipolysis through the cAMP–PKA–perilipin/HSL pathway (Xiong et al. 2015), and regulates adipose progenitor cells that give rise to beige fat through a CD81-integrin-FAK signaling axis (Oguri et al. 2020). In the liver, irisin promotes glycogenesis and suppresses gluconeogenesis and cholesterol synthesis through GSK3, FOXO1, and AMPK–SREBP2 pathways (Liu et al. 2015; Tang et al. 2016). It is important to emphasize, however, that majority of these multi-organ effects are derived from rodent or cell models; corresponding human mechanistic evidence is currently absent, and the integrin αVβ5 receptor framework itself has been validated principally in bone and adipose tissue.

In humans, acute aerobic exercise increases circulating irisin, although the magnitude varies across studies (Zugel et al. 2016; Fox et al. 2018; Cosio et al. 2023). The effects of chronic training are less consistent, with meta-analyses reporting heterogeneous or even opposing findings depending on exercise modality, duration, and study population (Qiu et al. 2015; Cosio et al. 2021; Mohammad Rahimi et al. 2022). This variability is compounded by a long-standing controversy over the detection and quantification of irisin in humans. Early studies questioned whether human FNDC5 could be efficiently translated given its non-canonical ATA start codon and whether ELISA-based irisin assays were reliable (Raschke et al. 2013), with subsequent work criticizing ELISA-based assays for limited specificity and cross-reactivity (Maak et al. 2021). However, a human plasma tandem mass spectrometry study detected and quantified circulating irisin and showed an exercise-associated increase, supporting its biological existence (Jedrychowski et al. 2015). More recent evidence that FNDC5 can be translated from an upstream ATG start codon in humans further weakens the argument that human irisin is merely an artifact (Witmer et al. 2024).

Overall, irisin/FNDC5 is supported by substantial cell- and animal-based evidence for roles in metabolic regulation, muscle preservation, and muscle-organ crosstalk. Human studies support exercise responsiveness and biomarker potential, but the current literature remains limited by the lack of causal intervention studies, uncertainty regarding the tissue origin of circulating irisin, and unresolved measurement issues. Accordingly, irisin is best regarded at present as a compelling preclinical mediator and a plausible human biomarker or candidate mediator.

### Myostatin

Myostatin, a transforming growth factor (TGF)-β family member, is one of the best-established negative regulators of skeletal muscle growth (Mcpherron et al. 1997), and its expression is physiologically suppressed by exercise (Huang et al. 2025). Its role is supported by converging genetic evidence across species: myostatin deletion causes marked muscle hypertrophy in animals, and a child with a loss-of-function MSTN mutation showed pronounced muscularity (Schuelke et al. 2004). A recent multi-cohort study of 1.1 million individuals further showed that function-disrupting MSTN variants associate with greater muscle mass and strength and lower adiposity (Herman et al. 2026). These findings support myostatin as a causal regulator of muscle mass in both animals and humans.

Mechanistically, cell and animal studies have shown that myostatin induces muscle wasting and inhibits regeneration by binding ActRII receptors, with subsequent recruitment of type I receptors such as ALK4/5 and activation of Smad2/3 signaling, as well as non-canonical pathways including Wnt, Notch, MAPK, and PI3K/Akt (Rodgers and Ward 2022). Accordingly, preclinical studies consistently demonstrate that blockade of myostatin itself or of the broader activin receptor axis produces robust skeletal muscle hypertrophy and attenuates muscle atrophy (Lee 2021). For example, in dystrophic mice, inhibition of myostatin signaling using dominant-negative ActRIIB enhanced myoblast proliferation and fusion through myogenic regulatory factor remodeling, thereby improving regeneration and increasing muscle mass (Fakhfakh et al. 2011). Myostatin is also antagonized extracellularly by follistatin, follistatin-like 3 (FSTL-3), and growth and differentiation factor–associated serum protein 1 (GASP-1) (Lee 2021). However, this pathway is not fully myostatin-specific, because ActRII-directed interventions also affect signaling by other TGF-β family ligands, including activins and GDF11 (Lach-Trifilieff et al. 2014; Morvan et al. 2017). Therefore, not all phenotypes observed after ActRII blockade can be attributed exclusively to myostatin.

Beyond its control of muscle mass, myostatin influences whole-body metabolic homeostasis through effects on both muscle and adipose tissue. In skeletal muscle, myostatin inhibition in mice enhances muscle metabolic homeostasis by enhancing glucose uptake and insulin sensitivity, in part through GLUT1/4 activation (Abati et al. 2022), and in obese mice it improves glucose disposal and fatty acid metabolism independently of muscle hypertrophy, apparently through effects on mitochondrial gene translation (Eilers et al. 2020). In adipose tissue, myostatin inhibition reduces obesity and insulin resistance through enhanced lipolysis and mitochondrial lipid oxidation (Zhang et al. 2012), and in preadipocytes it inhibits adipogenesis through miR-124-3p, which targets the glucocorticoid receptor (Liu et al. 2019b). In humans, carriers of function-disrupting MSTN variants show lower adiposity (Herman et al. 2026), although in humans it remains difficult to separate direct effects of reduced myostatin signaling in adipose tissue from secondary effects mediated by altered muscle mass, energy expenditure, or physical performance.

These benefits come with important caveats. Although myostatin deficiency or inhibition generally increases muscle mass, animal studies have also reported secondary trade-offs, including impaired mitochondrial bioenergetics, reduced ATP production, and lower tricarboxylic acid cycle enzyme activity in myostatin-knockout mice (Gu et al. 2022; Wang et al. 2022a). Greater muscle size therefore does not necessarily translate into improved muscle quality or oxidative function, underscoring that hypertrophy, mitochondrial fitness, and functional performance are not always tightly coupled. Consistent with this, preclinical studies indicate that myostatin inhibition can increase muscle mass without proportionate improvement in muscle quality unless combined with resistance exercise (Jang et al. 2021).

From a translational standpoint, myostatin remains one of the most advanced muscle-derived signaling pathways, yet its translation to humans faces both interpretive and functional challenges. At the biomarker level, circulating myostatin in humans varies with sex and age, with lower levels in young women than in age-matched men, a peak in men during their twenties followed by age-related decline, and relatively stable levels across age groups in healthy women (Schafer et al. 2016). However, its associations with age, adiposity, and functional status are not consistent across cohorts (Planella-Farrugia et al. 2019; Wilhelmsen et al. 2024). Circulating myostatin may therefore be more informative as a contextual biomarker than as a direct surrogate of pathway activity. At the interventional level, pharmacologic targeting of myostatin or the ActRII pathway has reproducibly increased lean mass or thigh muscle volume. For example, the anti-myostatin antibody LY2495655 increased appendicular lean mass in older weak fallers and improved selected performance measures in a phase 2 trial (Becker et al. 2015). The ActRII-blocking antibody bimagrumab increased thigh muscle volume and lean mass in older adults with sarcopenia and showed benefit in some mobility-related endpoints, particularly in slower walkers (Rooks et al. 2017). Later randomized data, however, showed that despite favorable changes in body composition, functional improvement was less consistent than expected (Rooks et al. 2020). More recent phase 3 readouts in spinal muscular atrophy further illustrate the variability of clinical outcomes for myostatin/ActRII-directed agents: the SAPPHIRE trial of the selective anti-myostatin antibody apitegromab met its primary motor-function endpoint when added to SMN-targeted therapy (Crawford et al. 2025), whereas the RESILIENT phase 3 trial of taldefgrobep alfa, an anti-myostatin adnectin, did not meet its primary endpoint despite robust target engagement (Servais et al. 2024). These contrasting outcomes reinforce that target engagement alone is not sufficient to predict clinical benefit. The mass–function disconnect likely reflects the fact that myostatin/ActRII inhibition primarily increases muscle mass, whereas physical performance in older adults also depends on muscle quality, neuromuscular function, and rehabilitation context. Overall, myostatin is a central regulator of muscle mass control and one of the most clinically advanced myokine-related targets, although its broader functional benefits in humans are not yet uniformly established.

### Growth differentiation factor 11

GDF11, also known as bone morphogenetic protein 11 (BMP11), is a member of the TGF-β superfamily (Zhang et al. 2018) that has attracted attention as an exercise-responsive myokine. Its interpretation as a circulating factor is complicated by contributions from multiple tissues and are highly sensitive to assay specificity, particularly cross-reactivity with myostatin-related epitopes (Egerman et al. 2015; Schafer et al. 2016). Despite these methodological caveats, both human observational and animal exercise studies have linked GDF11 to exercise-related phenotypes. In older adults, lifelong exercise was associated with higher circulating GDF11 levels, which correlated positively with peak muscle power, suggesting a role for GDF11 in exercise-mediated healthy aging (Elliott et al. 2017). Consistent with this, treadmill exercise in mice selectively increased GDF11 mRNA expression in slow-twitch muscles (Lee et al. 2019).

As a close homolog of myostatin, GDF11 has emerged as one of the most controversial proposed “rejuvenation” factors in the field. Although GDF11 and myostatin share strong structural and biochemical similarity, and plausibly engage a related ActRII-like receptor-SMAD2/3 signaling framework (Tsuchida et al. 2008), the physiological consequences of GDF11 signaling remain far less settled than those of myostatin (Suh and Lee 2020). An initial report suggested that restoring GDF11 in aged mice improved skeletal muscle structure and strength and enhanced stem cell genomic integrity (Sinha et al. 2014). A later study challenged both the direction and interpretation of these findings, showing instead that GDF11 increases with age and inhibits skeletal muscle regeneration by suppressing satellite cell expansion and myoblast differentiation (Egerman et al. 2015).

Subsequent mechanistic work has largely supported a catabolic role for GDF11 in muscle. In cell and animal models, GDF11 overexpression induces cardiac and skeletal muscle atrophy through activation of Smad2/3 signaling, leading to FOXO1 engagement and upregulation of the ubiquitin–proteasome system and autophagy pathways (Zimmers et al. 2017; Honda et al. 2022). These findings are directionally opposite to the original rejuvenation narrative and suggest that excessive or prolonged GDF11 signaling is catabolic in muscle. Consistent with this interpretation, administration of a GDF11 propeptide–Fc fusion protein promoted skeletal muscle hypertrophy and improved muscle strength in mdx mice (Jin et al. 2019). However, the propeptide-Fc construct also inhibits myostatin, so this result does not isolate a GDF11-specific effect.

Beyond skeletal muscle, recent studies have explored roles for GDF11 in bone, adipose tissue, and systemic metabolism. In bone, GDF11 has been linked to osteogenic processes, yet treatment with a dual myostatin/GDF11 inhibitor increased muscle mass while compromising bone integrity, again highlighting the difficulty of interpreting this pathway when closely related ligands are manipulated simultaneously (Suh et al. 2020). In adipose tissue and obesity models, GDF11 has been reported to improve glucose and insulin homeostasis by reducing white adipocyte size and enhancing glucose uptake in mature adipocytes (Frohlich et al. 2022), and to inhibit adipogenesis in preadipocytes via the Wnt/β-catenin and Smad2/3 pathways (Lin et al. 2023). GDF11 improves insulin sensitivity and energy expenditure in obesity through pathways including PI3K/Akt, AMPK, and TGF-β/Smad2 signaling (Lu et al. 2019).

Human evidence remains limited and does not support a simple “youth factor” model. Although lifelong exercise has been associated with higher circulating GDF11 in older adults (Elliott et al. 2017), quantitative human assay-development studies have suggested that circulating GDF11 may actually trend upward with age, directly challenging the original notion that age-related decline in GDF11 explains functional deterioration (Schafer et al. 2016). More broadly, human data are constrained by the fact that many earlier immunoassays lacked sufficient specificity to distinguish GDF11 from closely related ligands, particularly myostatin, thereby complicating interpretation of age-related or disease-related trends (Egerman et al. 2015; Schafer et al. 2016). Thus, the current human literature provides, at most, observational support for an association between GDF11 and healthy aging phenotypes, but does not establish GDF11 as a causal mediator of exercise adaptation in humans.

### IL-15

IL-15 belongs to the IL-2 superfamily and is primarily known for its anabolic effects on skeletal muscle (Quinn et al. 1995). Although skeletal muscle is an important source of IL-15, circulating levels may also reflect contributions from immune cell populations, complicating the interpretation of serum or plasma measurements in exercise studies (Duan et al. 2024).

In humans, exercise regulates IL-15 in a protocol-dependent manner. A recent meta-analysis showed that acute exercise generally increases circulating IL-15, whereas the effects of chronic training effects are more heterogeneous (Khalafi et al. 2023). High-intensity exercise, particularly under hypoxic conditions, increases IL-15 protein expression in skeletal muscle and is associated with reduced oxygen deficit and STAT3 activation (Pérez-López et al. 2019). Resistance exercise elevated serum IL-15 and activated the IL-15/IL-15Rα axis in human skeletal muscle, with associations to myofibrillar protein synthesis (Pérez-López et al. 2018). These findings support IL-15 responsiveness to exercise in humans, but the current evidence remains largely acute or correlational.

Mechanistically, IL-15 signals through a well-defined receptor system consisting of IL-15Rα together with IL-2Rβ and the common γ chain (γc). Notably, IL-15Rα not only binds IL-15 with high affinity but also regulates its stability, bioavailability, and trans-presentation (Budagian et al. 2006), making receptor biology a key determinant of IL-15 action. Downstream of this receptor system, cell and animal studies support direct metabolic actions of IL-15 in skeletal muscle. IL-15 enhances glucose uptake in myocytes in vitro and skeletal muscle in vivo by activating the JAK3/STAT3 pathway (Krolopp et al. 2016), and improves glucose tolerance by increasing GLUT4 translocation and AMPK phosphorylation (Fujimoto et al. 2019). Additional cell-based studies showed that IL-15 enhances mitochondrial activity through PPARδ-dependent signaling (Thornton et al. 2016), while protecting myotubes against H_2_O_2_-induced oxidative stress (Li et al. 2014). Beyond these metabolic effects, recent animal studies suggest that IL-15 also contributes to muscle quality and regeneration. IL-15 knockout mice exhibit reduced autophagy, whereas transgenic mice overexpressing IL-15 show improved muscle quality despite reduced muscle mass (Tanaka et al. 2024), indicating that IL-15 may influence muscle function in ways not fully captured by muscle size alone. Moreover, IL-15 has been implicated in the regulation of fibro-adipogenic progenitors (FAPs) during muscle injury, promoting their proliferation while suppressing adipogenic differentiation (Kang et al. 2018). These findings suggest that IL-15 extends beyond classic anabolic signaling and may also shape the regenerative microenvironment after muscle injury.

Beyond skeletal muscle, IL-15 has been linked to muscle-fat crosstalk and whole-body metabolic regulation. Cell and animal studies have shown that IL-15 reduces adipocyte differentiation and WAT mass while enhancing adiponectin secretion (Fuster et al. 2011), supporting its anti-obesity role. In line with this, muscle-derived IL-15 signaling contributes to protection against diet-induced obesity, and muscle-specific deletion of the IL-15 receptor in mice impairs IL-15 secretion and attenuates this protective effect (Zumbaugh et al. 2021). However, metabolic outcomes of IL-15 manipulation differ across models. In contrast to muscle-specific loss-of-function data, global *Il15ra*-null mice have also been reported to show increased oxidative metabolism and resistance to diet-induced obesity (Loro et al. 2015). This discrepancy suggests that outcomes depend on whether the intervention affects muscle-specific secretion, systemic receptor biology, or broader non-muscle tissues. Thus, although preclinical studies support a role for IL-15 in body weight regulation and muscle-adipose communication, the direction and mechanism of effect appear to be model dependent.

Translating these preclinical findings to humans requires further caution because IL-15 action is dose- and exposure-dependent. In skeletal muscle, acute IL-15 exposure enhances glucose uptake and mitochondrial activity, whereas chronic exposure can produce biphasic effects on mitochondrial respiration, with increased oxygen consumption observed only at low and very high concentrations (Nadeau et al. 2019). This non-linear response has important translational implications, as transient exercise-induced increases in IL-15 are unlikely to be equivalent to chronic pharmacologic elevation. Together with the model-dependence noted above, the beneficial effects associated with exercise-induced IL-15 therefore cannot be assumed to translate directly to systemic IL-15 therapy, particularly given the broader immunologic pleiotropy of IL-15.

Consistent with these preclinical uncertainties, human evidence for IL-15 remains supportive but incomplete. Acute resistance exercise in humans clearly activates the IL-15/IL-15Rα pathway in muscle and increases circulating IL-15 (Pérez-López et al. 2018), and broader exercise studies support an acute rise in circulating IL-15 after exercise (Khalafi et al. 2023). However, the literature remains heterogeneous with respect to exercise modality, sampling window, training status, obesity status, and the compartment measured. Moreover, because circulating IL-15 is not muscle specific and may also reflect immune-cell biology, the extent to which the exercise-induced signal originates from contracting skeletal muscle remains unclear. Thus, IL-15 is regarded as an exercise-responsive factor with a credible receptor axis and consistent preclinical metabolic effects, but not yet at the level of human causal evidence available for IL-6 or myostatin.

### Brain-derived neurotrophic factor

BDNF is a neurotrophin widely recognized for its critical roles in neuronal growth, survival, and differentiation (Azman and Zakaria 2022), and has been proposed as an exercise-responsive myokine. However, its classification as a *bona fide* endocrine myokine remains contested, and muscle-derived BDNF acts predominantly through autocrine and paracrine rather than endocrine signaling. Meta-analyses of randomized controlled trials have shown that physical exercise increases circulating BDNF levels in humans (Wang et al. 2022b), but this rise does not appear to originate primarily from skeletal muscle. Early human arteriovenous studies found no net BDNF release from exercising muscles despite increased muscular expression (Matthews et al. 2009), whereas arteriovenous sampling across the brain identified it as the dominant source of plasma BDNF during prolonged exercise (Rasmussen et al. 2009). More recent isoform-specific studies further showed that human skeletal muscle predominantly expresses pro-BDNF rather than mature BDNF, and that exercise-induced increases in serum mature BDNF are more consistent with platelet-related release than with direct muscular secretion (Edman et al. 2024; Tarassova et al. 2025).

Despite this uncertainty at the circulating level, cell and animal studies support a credible local role for BDNF within skeletal muscle. In cell models, muscle contraction induces BDNF expression, and BDNF enhances fatty acid oxidation through AMPK activation, consistent with this local metabolic role (Matthews et al. 2009). Animal studies have further implicated BDNF in muscle fiber specification and metabolic remodeling. Overexpression of BDNF in mice shifts muscles toward a more glycolytic phenotype, whereas BDNF deficiency increases fatigue resistance and the expression of slow muscle genes (Delezie et al. 2019). More recent animal studies have extended these observations by providing stronger causal evidence for a local metabolic role of muscle-derived BDNF. BDNF regulates mitochondrial dynamics, including fission and mitophagy, through the AMPK–PINK1–Parkin and AMPK–DRP1–MFF pathways; in muscle-specific BDNF knockout mice, loss of BDNF disrupts mitochondrial function and leads to metabolic dysfunction and obesity, whereas BDNF mimetics increase mitochondrial content and enhance mitofission and mitophagy in skeletal muscle (Ahuja et al. 2022). Muscle-derived BDNF is required for post-exercise recovery, as muscle-specific BDNF knockout mice show reduced activation of PPARδ-dependent metabolic genes, decreased intramuscular lipid use, delayed recovery after exercise, and impaired gains in endurance capacity (Chan et al. 2024). In female mice, muscle-specific BDNF deficiency impaired the switch from carbohydrate to fat utilization and promoted insulin resistance and muscle pathology; these effects were much less apparent in males, indicating a sex-dependent role (Yang et al. 2019). Of note, these mechanistic insights are derived mostly from genetically modified mice and cell models and have not yet been corroborated in human studies.

A defined receptor axis strengthens the mechanistic plausibility of these effects. BDNF signals through the TrkB receptor (NTRK2), an established pathway in both neural and peripheral tissues (Huang and Reichardt 2003). Beyond canonical TrkB signaling, the TrkB.T1 isoform expressed in pancreatic β-cells mediates a peripheral metabolic effect of BDNF on insulin secretion, and muscle-specific BDNF knockout mice recapitulate the impaired glucose tolerance and reduced insulin secretion observed after β-cell–specific TrkB.T1 deletion (Fulgenzi et al. 2020). This phenocopy provides evidence that muscle-derived BDNF can influence a distant metabolic tissue through a paracrine-like mechanism.

The possible role of BDNF in muscle-brain communication has also attracted interest. In rodent studies, exercise increases hypothalamic BDNF expression (Takimoto 2014), and FNDC5 overexpression elevates circulating irisin and enhances hippocampal BDNF expression (Wrann et al. 2013). These findings support the broader concept that exercise-induced muscle signals can influence brain neuroplasticity. Whether muscle itself contributes meaningfully to this brain BDNF signal, however, remains unresolved in light of the source-attribution issues noted above.

A major methodological challenge in the BDNF field is that circulating measurements are highly sensitive to sample handling. In particular, serum and plasma reflect different biological pools, as serum BDNF rises during clotting whereas plasma BDNF is strongly influenced by platelet removal and processing conditions (Gejl et al. 2019). This complexity is compounded by substantial inter-assay variability and differences in the detection of mature BDNF versus pro-BDNF (Polacchini et al. 2015). Accordingly, apparent increases in circulating BDNF after exercise should be interpreted cautiously unless sample type, processing conditions, and isoform specificity are clearly defined, reinforcing the view that BDNF is regarded as a credible local mediator and exercise-associated biomarker rather than an established endocrine myokine.

### Meteorin-like

METRNL, also known as subfatin, is an exercise- and cold-responsive secreted factor that has emerged as a candidate myokine linking skeletal muscle activity to immunometabolic remodeling. It was first identified by Rao et al. as a PGC1α4-associated factor induced in skeletal muscle after exercise and in adipose tissue after cold exposure. In the same study, increasing circulating METRNL promoted beige-fat thermogenesis, enhanced energy expenditure, and improved glucose tolerance through eosinophil-dependent IL-4/IL-13 signaling and alternative macrophage activation (Rao et al. 2014). Thus, METRNL emerged as a mediator of immune–adipose crosstalk, with subsequent studies extending its reported actions to skeletal muscle and other metabolic tissues.

Among these, muscle-related actions have been most extensively characterized. In mice, METRNL facilitates muscle repair by promoting macrophage transition toward an anti-inflammatory phenotype through a Stat3/IGF-1 mechanism and by supporting satellite-cell proliferation (Baht et al. 2020). Whole-body and macrophage-specific METRNL deficiency impaired regeneration, whereas parabiosis restored muscle repair, suggesting that injured muscle METRNL derives in part from infiltrating immune cells (Baht et al. 2020). Consistent with a broader role in tissue remodeling, METRNL enhances regeneration in aged muscle by counteracting pro-fibrotic programs and by promoting apoptosis of FAPs (Lee et al. 2022a).

METRNL also influences skeletal muscle insulin sensitivity and whole-body metabolic control. In high-fat diet (HFD)-fed mice, METRNL alleviates lipid-induced inflammation and insulin resistance in skeletal muscle via AMPK- or PPARδ-dependent pathways (Jung et al. 2018a), and improves glucose tolerance in skeletal muscle cells and mice through AMPKα2-dependent signaling (Lee et al. 2020). In obesity, METRNL reduces IL-1β secretion in macrophages and enhances insulin sensitivity via PPARγ activation (Javaid et al. 2021). However, METRNL overexpression inhibits adipogenesis and promotes proliferation in stromal vascular fraction cells, with accompanying insulin resistance (Löffler et al. 2017).

Beyond skeletal muscle and adipose tissue, animal studies have also implicated METRNL in cardiovascular protection. METRNL protects against cardiac hypertrophy and dysfunction through AMPK and SIRT1-related pathways, reduces doxorubicin-induced cardiotoxicity, and promotes post-infarction angiogenesis (Hu et al. 2020; Reboll et al. 2022; Cao et al. 2024). In this cardiovascular setting, a major mechanistic advance came with the identification of the KIT receptor tyrosine kinase as a high-affinity METRNL target. In cultured human endothelial cells and mouse myocardial infarction models, METRNL promotes angiogenesis and infarct repair through KIT-dependent signaling, whereas METRNL deficiency impairs this response (Reboll et al. 2022). Whether KIT also mediates the metabolic effects of METRNL across skeletal muscle, adipose tissue, and immune cells remains unresolved. These cardiovascular actions are at present confined to rodent infarction and hypertrophy models, and no human studies have yet linked circulating METRNL to cardioprotective clinical outcomes.

In human skeletal muscle, high-intensity interval exercise increases METRNL mRNA expression (Eaton et al. 2018), and acute human exercise studies showed that downhill running increased circulating METRNL together with eosinophil counts, consistent with the immunoregulatory framework proposed in preclinical models (Alizadeh and Alizadeh 2022). More recently, a 12-week randomized controlled exercise study in older adults found that serum METRNL increased after walking-based exercise interventions and that these changes correlated with improvements in inflammatory markers and physical function (Jamrasi et al. 2025). Human disease-association data, however, are heterogeneous. One large clinical study reported that circulating METRNL was higher in people with type 2 diabetes and was associated with fasting glucose, blood pressure, lipid variables, and renal function (Chung et al. 2018). A later meta-analysis, however, found no overall difference in circulating METRNL between patients with type 2 diabetes and controls, with subgroup results varying by BMI, age, HOMA-IR, sample type, and sample size (Wu et al. 2020). Taken together with the preclinical data, METRNL is regarded as a candidate immunometabolic mediator and biomarker in human exercise biology.

### Secreted protein acidic and rich in cysteine

SPARC, also known as osteonectin, is a matricellular glycoprotein involved in extracellular matrix remodeling, fibrosis, and metabolic regulation. SPARC secretion increases after acute exercise in the skeletal muscle of mice and in the circulation of healthy young adults, suggesting that it may participate in exercise-induced muscle adaptation (Aoi et al. 2013; Reichel et al. 2023). However, the human literature is not fully consistent, as a sprint study found no increase in circulating SPARC after either acute or chronic supramaximal exercise (Songsorn et al. 2017). This suggests that the SPARC response to exercise is protocol dependent and may vary with exercise modality, intensity, sampling conditions, and metabolic status.

Within skeletal muscle, SPARC binds directly to actin in regenerating myofibers, contributing to force recovery after fatigue, whereas SPARC-deficient mice exhibit impaired post-fatigue muscle recovery (Jørgensen et al. 2017). In addition, SPARC enhances glucose tolerance and skeletal muscle glucose uptake through AMPK activation, thereby mimicking some of the metabolic benefits of exercise (Aoi et al. 2019). Moreover, SPARC-induced AMPK activation was calcium dependent and associated with strong interaction with a voltage-dependent calcium channel, suggesting a plausible, albeit incomplete, target framework for its metabolic effects (Aoi et al. 2019). These findings support SPARC as a plausible preclinical mediator of muscle metabolic adaptation, although a clearly defined canonical receptor axis has not yet been established.

SPARC has also been implicated in aging-related skeletal muscle decline. In mice, SPARC exerts protective effects during aging, as SPARC overexpression preserves muscle strength and mitochondrial function, increases glucose transporter and oxidative phosphorylation-related protein expression, and lowers glycemia and adiposity, whereas SPARC deficiency recapitulates aging-associated phenotypes (Ghanemi et al. 2022). In parallel, IGF-I stimulates SPARC expression through PI3K signaling, potentially limiting age-related intramuscular fat accumulation (Mathes et al. 2022).

Beyond skeletal muscle, SPARC participates in broader metabolic crosstalk, particularly in adipose biology, although its effects appear to be context-dependent. At the cellular level, SPARC suppresses adipogenesis via Wnt/β-catenin signaling (Nie and Sage 2009). In aged muscle, however, the FGF2/miR-29a/SPARC axis instead promotes intramuscular adipogenesis (Mathes et al. 2021). In humans, circulating SPARC levels are elevated in obesity and correlate with reduced lipid storage capacity (Atorrasagasti et al. 2023), and acute circulating myokine responses, including SPARC, may differ by obesity status in women (Garneau et al. 2020).

SPARC biology is further complicated by its pleiotropic roles in inflammation and tissue remodeling. SPARC activates the NLRP3 inflammasome, whereas SPARC inhibition mimics caloric restriction by increasing energy expenditure and lowering inflammation (Ryu et al. 2023). SPARC has also been linked to metabolic dysfunction-associated steatotic liver disease (MASLD) (Mazzolini et al. 2018; Onorato et al. 2021), and its upregulation contributes to pulmonary hypertension (Veith et al. 2022). Conversely, exercise-associated reductions in SPARC have been associated with improved lipid metabolism and vascular function in some settings (Hu et al. 2024).

From a translational perspective, SPARC remains at an early stage. The strongest support comes from cell and animal studies, including evidence that recombinant SPARC rescues metabolic defects in SPARC-deficient mice and enhances AMPK-dependent glucose uptake in skeletal muscle (Aoi et al. 2019), and that a single intraperitoneal injection of recombinant SPARC is sufficient to elicit exercise-like phenotypic responses in mice (Ghanemi et al. 2025). Although acute aerobic exercise can increase circulating SPARC in humans (Aoi et al. 2013; Reichel et al. 2023), no mechanistic human studies have yet shown that selective SPARC modulation improves metabolic outcomes or exercise adaptation, and its broad tissue expression and the absence of a clearly defined dominant receptor continue to limit therapeutic interpretation. Accordingly, SPARC is best regarded at present as a biologically plausible but pleiotropic myokine, with stronger support as a local or preclinical mediator than as a clinically established human exercise factor.

### Fibroblast growth factor 21

FGF21 is a stress-responsive hormone-like factor classically recognized as a hepatokine produced by the liver during fasting and other metabolic stress states (Fisher and Maratos-Flier 2016), but it also functions as a myokine under specific metabolic and mitochondrial stress conditions in skeletal muscle (Izumiya et al. 2008). Because hepatic production remains the dominant endocrine source in many physiological and pathological settings, however, exercise-induced increases in circulating FGF21 do not by themselves establish skeletal muscle as the principal source. In humans, circulating FGF21 levels are elevated in obesity and type 2 diabetes and correlate with fasting insulin and body mass index, generally interpreted as a compensatory response to metabolic stress rather than as a direct myokine signal (Mashili et al. 2011). FGF21 is therefore viewed as a stress-inducible hepatokine that can also act as a myokine.

FGF21 signals through a relatively well-defined receptor axis, requiring β-Klotho as a coreceptor and acting primarily through FGFR1c, with FGFR3c also contributing in certain cellular contexts (Suzuki et al. 2008). This clear receptor biology has made the FGF21 pathway one of the most pharmacologically tractable among exercise-associated factors, as discussed below. Within skeletal muscle, FGF21 is induced under specific conditions, but whether it drives adaptation or merely marks stress remains debated. Electrical stimulation studies showed that FGF21 expression and secretion are induced in skeletal muscle through PI3K/Akt/mTOR signaling (Arias-Calderón et al. 2023), and cell-based work demonstrated that FGF21 enhances muscle glucose uptake via the PI3K/PKC-ζ and GLUT4 pathways (Rosales-Soto et al. 2020). Additional animal studies implicated FGF21 in fasting-induced muscle atrophy through Bnip3-mediated mitophagy (Oost et al. 2019) and endurance exercise-induced fiber-type conversion through TGF-β1 and p38 MAPK signaling (Luo et al. 2023). However, the role of endogenous muscle-derived FGF21 in mitochondrial adaptation is contested. One study in mice and humans with mitochondrial disorders reported that skeletal muscle increased FGF21 expression as a compensatory response and that FGF21 enhanced mitochondrial function through an mTOR–YY1–PGC1α–dependent pathway in muscle cells (Ji et al. 2015). Another study, however, concluded that muscle mitochondrial stress adaptation operates largely independently of endogenous FGF21 action (Ost et al. 2016). Thus, FGF21 contributes to muscle metabolic remodeling, but its expression may also reflect mitochondrial stress or catabolic pressure.

Beyond skeletal muscle, FGF21 exerts broad systemic metabolic actions that are much better established than its muscle-specific role. Early cell and animal studies established FGF21 as a potent metabolic regulator, showing that recombinant FGF21 increases glucose uptake in adipocytes, improves glycemia and triglyceride levels in diabetic rodents, and protects against diet-induced obesity without classical mitogenic effects (Kharitonenkov et al. 2005). Consistent with these effects, FGF21 promotes hepatic fatty acid oxidation, stimulates lipolysis, and supports browning of WAT (Itoh 2014; Fisher and Maratos-Flier 2016). FGF21 also protects against diabetic cardiomyopathy and myocardial infarction through the AMPK/FOXO3/SIRT3 and anti-fibrotic pathways (Ma et al. 2021; Jin et al. 2022), reduces vascular pyroptosis in exercise settings (Li et al. 2022), and activates hepatic lipophagy to improve MASLD (Gao et al. 2020). In obese mice, short-term FGF21 administration reduced body weight in both sexes without changing total energy intake, although downstream neuroendocrine and metabolic outcomes differed between males and females, including sex-specific changes in adipose tissue gene expression (Makarova et al. 2021).

Human evidence reinforces this distinction between pharmacologic readiness and exercise-specific mechanistic certainty. A recent meta-analysis confirmed that exercise influences circulating FGF21 levels in humans, but the direction and magnitude of the change depend on exercise modality, duration, and population studied. Indeed, longer-duration or concurrent training may even reduce circulating FGF21 in adults with metabolic disorders (Kim et al. 2023). By contrast, pharmacologic intervention provides much stronger causal human evidence. A randomized placebo-controlled trial of the FGF21 analogue LY2405319 in obese adults with type 2 diabetes demonstrated improvements in dyslipidemia, body weight-related measures, fasting insulin, and adiponectin, albeit with only a trend toward glucose lowering (Gaich et al. 2013). More recently, the phase 2b pegozafermin trial showed histologic improvement in fibrosis and metabolic dysfunction-associated steatohepatitis (MASH)-related endpoints in biopsy-proven noncirrhotic MASH, supporting continued phase 3 development (Loomba et al. 2023). FGF21 is therefore regarded as a metabolic hormone with inducible myokine features, with its receptor pathway clearly druggable and clinically active in humans, but its role as a direct mediator of exercise adaptation less certain.

### β-aminoisobutyric acid

BAIBA, a product of valine and thymine catabolism, has emerged as an exercise-responsive small-molecule myometabolite with broad metabolic effects (Yi et al. 2023). In the original cell, animal, and human translational study, BAIBA was identified as a PGC-1α-associated metabolite released from myocytes that induces thermogenic gene programs in white adipose tissue, stimulates hepatic β-oxidation, improves glucose homeostasis in mice, and is inversely associated with cardiometabolic risk factors in humans (Roberts et al. 2014). Unlike peptide myokines, however, BAIBA exists as distinct D- and L-enantiomers with different metabolic origins and potentially different biological properties. In an enantiomer-resolved acute exercise crossover study, both D- and L-BAIBA increased after exercise, but baseline levels of the D-enantiomer were far higher and strongly influenced by AGXT2 genotype (Stautemas et al. 2019). BAIBA is therefore an exercise-responsive myometabolite whose interpretation requires attention to both stereochemistry and host genetic background.

In skeletal muscle, cell and animal studies indicate that BAIBA enhances mitochondrial fatty acid oxidation, improves insulin signaling through the IRS-1/Akt and AMPK–PPARδ pathways, and exerts anti-inflammatory effects (Roberts et al. 2014; Jung et al. 2015). These findings support BAIBA as a plausible preclinical mediator of muscle-associated metabolic adaptation. In the adipose tissue, animal and adipocyte studies indicate that BAIBA increases fatty acid oxidation, reduces lipid accumulation, and induces browning-related thermogenic genes such as UCP1, thereby enhancing energy expenditure (Roberts et al. 2014). Additional adipocyte studies showed that BAIBA improves insulin sensitivity through AMPK and IRS-1 signaling and suppresses inflammatory pathways including NF-κB, further supporting an anti-inflammatory, insulin-sensitizing role in adipose tissue (Jung et al. 2018b). In the liver, mouse and hepatocyte studies indicate that BAIBA stimulates β-oxidation and alleviates hepatic endoplasmic reticulum stress, apoptosis, and glucose/lipid metabolic disturbance through AMPK-dependent mechanisms (Roberts et al. 2014; Shi et al. 2016).

BAIBA has also emerged as a candidate mediator of muscle-bone crosstalk. Circulating L- and D-BAIBA levels are differentially associated with body composition, bone density, and functional performance in humans (Lyssikatos et al. 2023), suggesting enantiomer-specific effects on the musculoskeletal system. At the cellular level, L-BAIBA protects osteocytes from oxidative stress–induced death through MRGPRD signaling, supported by antagonist, knockdown, and knockout approaches (Kitase et al. 2018). L-BAIBA also promotes bone formation through Wnt, TGFβ, and BMP signaling (Prideaux et al. 2023). More recently, both BAIBA enantiomers have been shown to regulate FGF23 expression in osteocytes through MRGPRD but via distinct downstream pathways, with L-BAIBA acting directly through Gαs/cAMP/PKA and Gαq/PKC signaling and D-BAIBA acting indirectly via sclerostin (Sakamoto et al. 2024). These findings establish MRGPRD as a partially defined BAIBA receptor in bone, but whether the same axis mediates BAIBA action in skeletal muscle or other metabolic tissues remains unclear.

Human evidence remains supportive but limited. As noted above, circulating BAIBA increases with exercise in humans, and its two enantiomeric forms show distinct abundance patterns and genetic influences (Roberts et al. 2014; Stautemas et al. 2019). In addition, L- and D-BAIBA show different human phenotype associations, with L-BAIBA more closely linked to BMI and bone mineral density, and D-BAIBA to age and physical performance (Lyssikatos et al. 2023). Thus, human interpretation of BAIBA requires enantiomer-resolved measurement and consideration of host genotype. Accordingly, BAIBA is best viewed in humans as an exercise-associated biomarker or correlate of metabolic state, with mediator biology supported mainly by preclinical data.

### Leukemia inhibitory factor

LIF is a contraction-induced myokine that functions predominantly as a local autocrine/paracrine regulator of skeletal muscle rather than as a circulating endocrine factor. In humans, LIF mRNA is markedly induced in skeletal muscle after endurance and resistance exercise, whereas plasma LIF remains undetectable; consistently, electrically stimulated human myotubes secrete LIF in vitro (Broholm et al. 2012).

Mechanistically, LIF signals through the canonical LIFRβ-gp130 receptor complex, which activates JAK/STAT, MAPK, and PI3K-associated pathways. This relatively well-defined receptor axis provides strong mechanistic support for LIF biology, but it also underscores its pleiotropy, since LIFR/gp130 signaling can produce different outcomes depending on cell type and physiological setting (Nicola and Babon 2015). In human skeletal muscle, exercise-related PI3K-Akt-mTOR signaling contributes to LIF induction, and recombinant LIF promotes myoblast proliferation through JunB and c-Myc activation, with LIFR knockdown suppressing this response (Broholm et al. 1985). Similar proliferative effects extend to satellite cells through JAK2–STAT3 signaling (Spangenburg and Booth 2002), and more recent work continues to position LIF as a key exercise-induced regulator of satellite cell function relevant to muscle maintenance (Guo et al. 2024). Beyond proliferation, LIF acutely increases glucose uptake in mouse skeletal muscle through a PI3K/mTORC2/Akt-dependent mechanism, supporting a direct metabolic action in addition to its regenerative role (Brandt et al. 2015). In line with these combined effects, increased LIF and LIFR expression, together with STAT3 activation, has been observed after interval exercise training in rats and correlates with prevention of myocardial infarction–induced skeletal muscle atrophy (Jia et al. 2018). This regenerative capacity may be compromised in disease, as LIF-induced STAT1 and STAT3 phosphorylation and the associated proliferative response are impaired in myoblasts from patients with diabetes, suggesting a mechanism that may contribute to muscle dysfunction in diabetes (Broholm et al. 2012).

Beyond these muscle-intrinsic actions, LIF also exerts immunomodulatory effects in diseased muscle. In Duchenne muscular dystrophy (DMD) models, selective expression of LIF in leukocyte progenitors infiltrating dystrophic muscle suppressed Th2 cytokines, shifted macrophages away from a CD163⁺/CD206⁺ pro-fibrotic phenotype, and reduced TGF-β signaling and FAP activity (Welc et al. 2019). However, a subsequent study found that early localized overproduction of LIF causes macrophage aggregation and transient increases in muscle fiber damage (Flores et al. 2021), highlighting the importance of timing, spatial distribution, and dosage in determining LIF outcomes.

LIF has also been linked to interorgan physiology, but the evidence for a *bona fide* endocrine role remains limited. In bone, LIF enhances osteoblast differentiation and proliferation partly through STAT3 signaling (Sims and Johnson 2012), but these findings are more consistent with local cytokine action than with muscle-to-bone endocrine communication. This distinction is important because circulating LIF is difficult to detect reliably after exercise in humans, and the molecule has a very short circulating half-life in vivo, with early mouse pharmacokinetic studies reporting an initial half-life of approximately 6–8 min (Hilton et al. 1991). LIF is therefore a credible local mediator of muscle proliferation, regeneration, and glucose handling, rather than a validated circulating endocrine myokine, given its rapid clearance, poor post-exercise detectability, pleiotropy, and strong dependence on local tissue context. Taken together, the immunomodulatory, regenerative, and bone-related effects of LIF described above are established primarily in rodent and cell models, with corresponding human mechanistic evidence currently lacking.

### Apelin

Apelin, a peptide hormone produced by multiple tissues including skeletal muscle and adipose tissues, has emerged as an exercise-responsive myokine with important metabolic effects (Castan-Laurell et al. 2019). It signals through the APJ receptor (APLNR), a well-defined G protein-coupled receptor, to regulate cardiovascular function and energy metabolism (Hu et al. 2021). This relatively clear receptor axis strengthens the mechanistic plausibility of apelin as a myokine, although circulating apelin cannot be attributed exclusively to skeletal muscle given the broad tissue distribution of both the peptide and its receptor.

Apelin levels transiently increase after aerobic exercise and correlate with maximal oxygen uptake and cardiac function in humans and mice (Kon et al. 2022; Ligetvári et al. 2023). In humans, this acute response appears to be sex dependent, with a clearer post-exercise increase in men than in women (Son et al. 2019). In addition, endurance training increased skeletal muscle apelin expression in obese men, and the change in muscle apelin was associated with improved insulin sensitivity. Human primary myotubes also secreted apelin, supporting its classification as an exercise-regulated myokine with at least local autocrine/paracrine actions (Besse-Patin et al. 2014). These findings support exercise responsiveness in humans, although the available evidence remains largely associative and does not yet establish muscle-derived apelin as the dominant causal mediator of exercise adaptation in vivo.

Within skeletal muscles, apelin enhances glucose uptake, Akt phosphorylation, fatty acid oxidation, and mitochondrial oxidative capacity via AMPK, leading to improved lipid utilization and muscle insulin sensitivity in obese mice (Yue et al. 2010; Attané et al. 2012). Beyond these acute metabolic effects, apelin appears important for muscle adaptation to high-intensity exercise in rodent models, as apelin knockout mice exhibit reduced fiber size, impaired IGF-1 signaling, and altered fiber-type remodeling (Kilpiö et al. 2024). The role of apelin becomes particularly compelling in aging-related muscle decline, as apelin production decreases with age in both rodents and humans, whereas apelin supplementation in aged mice reverses age-related declines in muscle function by boosting mitochondrial content, enhancing autophagy, reducing inflammation, and improving muscle stem cell function and regeneration (Vinel et al. 2018). In this regard, the apelin–APJ system also attenuates CKD-induced muscle wasting (Enoki et al. 2023). At the cellular level, single-cell RNA sequencing and in vivo studies showed that apelin secretion is regulated by Tead1 in myogenic cells, whereas the apelin receptor is enriched in endothelial cells, indicating a paracrine crosstalk mechanism involved in muscle repair and vascular remodeling (Lee et al. 2022b). Together, these findings support apelin as a biologically relevant mediator of muscle adaptation, regeneration, and aging in preclinical models, acting both as a metabolic factor within myofibers and as a local organizer of the regenerative niche.

Systemically, apelin–APJ signaling contributes to thermogenesis and metabolic regulation in adipose tissue (Than et al. 2015), a mechanism proposed as one route underlying the beneficial effect of maternal exercise on offspring metabolic health (Son et al. 2022). Apelin also contributes to intestinal homeostasis, where exercise-induced APJ–AMPK activation supports mitochondrial biogenesis and fatty acid metabolism in the duodenal epithelium (Chae et al. 2023).

In humans, the apelin pathway is also supported by physiology and early pharmacology studies beyond biomarker associations. Apelin infusion has demonstrable physiological effects on the cardiovascular system (Japp et al. 2010), and APJ agonism has entered first-in-human testing (Winkle et al. 2023). These findings strengthen the clinical relevance of the apelin pathway, although direct exercise-mimetic trials targeting metabolic or musculoskeletal endpoints are still lacking, and cardiovascular safety considerations remain to be fully defined. Apelin is therefore a strong candidate mediator with meaningful clinical promise, supported by causal preclinical evidence but limited by unresolved tissue of origin and the absence of mechanistic human exercise trials.

### Musclin

Musclin, also known as osteocrin, is a skeletal muscle–enriched secreted peptide structurally related to natriuretic peptides. It was originally identified in mice as a muscle-derived factor whose expression is suppressed by fasting, induced by refeeding, and elevated in insulin-resistant states, where recombinant musclin impaired insulin-stimulated glucose uptake and glycogen synthesis in myocytes (Nishizawa et al. 2004).

A major advance came with the recognition of two facets of musclin biology, its receptor pathway and its exercise responsiveness. Mechanistically, musclin binds the natriuretic peptide clearance receptor NPR-C/NPR3, thereby modulating natriuretic peptide signaling (Moffatt et al. 2007). In parallel, musclin was subsequently identified as an activity-stimulated myokine, with exercise inducing its expression through Ca^2^⁺-dependent Akt activation and FOXO1 nuclear export in contracting muscle, and with secreted musclin then promoting endurance adaptation through ANP/cGMP–PGC1α signaling and mitochondrial biogenesis in skeletal muscle (Subbotina et al. 2015). In that study, convergent cell, animal, and human primary myoblast data showed activity-related musclin production, whereas musclin-disrupted mice exhibited reduced exercise tolerance, lower cGMP and PGC1α signaling, and reduced mitochondrial protein content, all of which were improved by recombinant musclin. These dual observations indicate that musclin action depends on physiological context, with contraction-induced musclin engaging adaptive pathways distinct from those associated with pathological metabolic states. Recent work has extended this adaptive role beyond endurance, as exercise-induced musclin reduces FAPs and fibrosis by promoting macrophage phagocytosis of apoptotic FAPs through a FILIP1L-dependent mechanism (Kang et al. 2024), and PGC1α-dependent musclin expression protects against cancer cachexia–induced muscle wasting (Re Cecconi et al. 2019).

Beyond skeletal muscle, musclin influences metabolic organs in divergent ways. In cell studies, musclin promotes lipolysis and suppresses lipogenesis via PKA/p38 signaling in 3T3-L1 adipocytes (Choi et al. 2023), and alleviates lipid accumulation and endoplasmic reticulum stress by upregulating SIRT7 and enhancing autophagy in primary hepatocytes (Cho et al. 2023). In contrast, in vivo studies showed that musclin is strongly temperature sensitive and suppresses beige-fat thermogenesis by binding transferrin receptor 1 (Tfr1) and inhibiting cAMP/PKA signaling, thereby worsening systemic energy homeostasis in male mice (Jin et al. 2023). This apparent discrepancy between cellular and whole-body effects, together with the identification of both NPR-C and Tfr1 as targets, suggests that the dominant receptor pathway and metabolic outcome of musclin may vary by tissue, sex, and ambient temperature.

Human evidence remains limited compared with the animal literature. The strongest human support currently comes from the original identification of musclin as a muscle-derived secretory factor, human primary myoblast data in the endurance study, and obesity-associated expression and circulating findings in the Tfr1 study (Nishizawa et al. 2004; Subbotina et al. 2015; Jin et al. 2023). However, no human perturbation studies have tested whether selective manipulation of musclin improves exercise performance, metabolic endpoints, or sarcopenia-related outcomes. Musclin is therefore a biologically plausible candidate mediator with strong preclinical support for endurance adaptation, muscle repair, and muscle–metabolic crosstalk, but not yet an established regulator in humans, given its conflicting phenotypes, sparse causal evidence, and sex- and temperature-dependent effects.

### Other myokines

In addition to the better-characterized myokines discussed above, global transcriptomic and secretome analyses continue to identify additional exercise-responsive candidates with potential metabolic relevance. Compared with IL-6, myostatin, or FGF21, most of these factors are currently supported mainly by acute human exercise signatures or preclinical functional studies, with limited human mechanistic evidence.

Decorin is released from skeletal muscles in response to acute resistance exercise in humans, and its expression also increases after chronic training in both mice and humans (Kanzleiter et al. 2014). Mechanistically, decorin promotes muscle hypertrophy through direct antagonism of myostatin, as indicated by increased expression of pro-myogenic factors and reduced atrogene expression in decorin-overexpressing mice. Decorin has also been implicated in muscle-pancreas crosstalk, because human islet studies showed that it increases insulin content and secretion and protects β-cells from inflammatory cell death (Langlois et al. 2025). These findings support plausible cross-tissue metabolic actions, although current human evidence remains limited to exercise responsiveness and ex vivo islet biology.

Follistatin, also secreted by skeletal muscle, is a biologically plausible anabolic myokine with a relatively well-defined myostatin/activin–ActRIIB target axis. In preclinical studies, follistatin promotes muscle anabolism and improves insulin action through AKT, p70S6K, TBC1D1/4, and PAK1 signaling (Lee et al. 2010; Han et al. 2019). However, circulating follistatin may include substantial contributions from non-muscle tissues, particularly the liver, where hepatic follistatin is increased in MASLD and has been reported to protect against steatosis through mTOR-related signaling (Tong et al. 2022). The endocrine contribution of muscle-derived follistatin in humans therefore remains insufficiently resolved, despite a credible mechanistic framework.

Follistatin-like 1 (FSTL1), structurally related to follistatin but functionally distinct, is an adipomyokine elevated after exercise and in type 2 diabetes (Xu et al. 2020). Recent cell-based evidence suggests that FSTL1 promotes lipid mobilization by stimulating glycerol release, cGMP production, and hormone-sensitive lipase activation via DIP2a (Nam et al. 2024), providing a plausible target mechanism. Whether exercise-induced FSTL1 functions primarily as a local paracrine factor, a systemic adipomyokine, or a biomarker of metabolic stress remains unresolved, reflecting the early stage of this literature.

Calprotectin, originally identified as a major cytosolic protein in neutrophils, is also released from human skeletal muscle during exercise (Mortensen et al. 2008). In humans, circulating calprotectin decreases with increasing training intensity and is associated with lower markers of inflammation and muscle damage (Bukvić et al. 2023), supporting its relevance as an exercise-responsive biomarker. However, its strong link to immune-cell biology implies that circulating calprotectin is unlikely to reflect skeletal muscle secretion alone, limiting its interpretation as a muscle-specific myokine.

## Pharmacological regulation of myokine synthesis and secretion

Given the therapeutic potential of the myokine pathways reviewed above, there is growing interest in natural products and small-molecule compounds that can modulate myokine expression or secretion independently of exercise, thereby offering a pharmacological route to mimic or complement the metabolic benefits of physical activity; recent examples are summarized in Table 3.

### Compounds regulating IL-6

Ouabain, a cardiotonic steroid naturally derived from *Strophanthus gratus*, increases IL-6 secretion from human skeletal muscle cells and decreases IL-6 signaling via proteasome-mediated degradation of STAT3 and activation of the ERK1/2 and Akt–mTOR pathways, suggesting negative feedback in the IL-6/STAT3 pathway (Pirkmajer et al. 2020). Caffeine, a methylxanthine alkaloid primarily found in coffee and tea, induces IL-6 mRNA expression and secretion in myotubes, but not in hepatocytes or adipocytes, via p38/MAPK signaling, leading to hepatic STAT3 activation and protection against MASLD in mice (Fang et al. 2019). Conversely, bazedoxifene, a clinically approved selective estrogen receptor modulator, inhibits IL-6/IL-6R/STAT3 signaling and exerts anti-inflammatory and anti-atherosclerotic effects in endothelial cells, vascular muscle cells, and apolipoprotein E-deficient mice (Luo et al. 2021).

### Compounds regulating irisin/FNDC5

Several compounds induce FNDC5 expression and irisin secretion, most commonly through PGC1α/AMPK signaling. Icariin, a prenylated flavonol glycoside and major bioactive component of the traditional Chinese herbal medicine, *Epimedii Herba*, activates the AMPK to enhance FNDC5 mRNA and protein expression in murine C2C12 myocytes and skeletal muscle (Chen et al. 2019). Pentamethylquercetin, a polymethoxylated flavonol derived from quercetin, and liraglutide increase circulating irisin and promote PGC1α and FNDC5 expression in murine myocytes, facilitating adipose tissue browning (Wu et al. 2022a; Zhang et al. 2024). Caffeine similarly promotes irisin secretion by enhancing PGC1α expression via calcium signaling in mouse skeletal muscle (Liu et al. 2023). FGF19 administration mitigates the decrease in FNDC5/irisin expression in palmitic acid–treated C2C12 myotubes and the skeletal muscle of HFD-fed mice by activating AMPK/SIRT1/PGC1α signaling (Guo et al. 2021). In contrast, sesamin, a plant-derived lignan abundantly found in sesame seeds, stimulates the SIRT1/SMAD3 axis to induce FNDC5 expression and irisin secretion in mouse skeletal muscle (Kou et al. 2022). Resveratrol, a polyphenolic compound with a stilbene structure found in grape skins and berries, and all-trans retinoic acid, an active derivative of vitamin A, synergistically upregulate FNDC5 expression in C2C12 cells (Abedi-Taleb et al. 2019). Aerobic training and ursolic acid, a pentacyclic triterpenoid present in plants, such as apple peel, increase FNDC5 gene expression in muscle and plasma irisin levels in rats (Teimourian et al. 2020). Quercetin, a flavonol abundantly present in fruits and vegetables including onions and apples, restores HFD-induced reductions in muscle FNDC5 and PGC-1α levels in rats partly through AMPK activation, raising plasma irisin and WAT browning markers (PRDM16, UCP-1, and PPARγ). In L6 myotubes, it similarly prevents palmitate-induced decreases in p-AMPK and FNDC5, and silencing *Pgc-1α* abolishes these effects, confirming PGC-1α dependence (Muscia Saez et al. 2026).

### Compounds regulating myostatin

Curcumin, a curcuminoid polyphenol derived from turmeric, suppresses myostatin and increases myogenin expression, reducing cachexia-related muscle atrophy in mice (Zhang et al. 2022). The green tea polyphenol, epigallocatechin-3-gallate (EGCG), inhibits elevated myostatin protein in late-passage C2C12 myoblasts and aged mice (Chang et al. 2020). *Glycyrrhiza uralensis* extract reduces myostatin, TNF-α, and MuRF1, restoring muscle histology in dexamethasone-induced sarcopenia in C2C12 myoblasts and mice (Zheng et al. 2024). In silico analysis showed that the *G. uralensis* components Licochalcone A and B bind myostatin and suppress its interaction with the receptor ActRIIB. This was confirmed experimentally by downregulation of myostatin, Atrogin1, and MuRF1 in C2C12 myoblasts and mice (Ahmad et al. 2024). A cedrol derivative, a bioactive sesquiterpene, suppresses myostatin transcription through Ca^2^⁺–CaMK–FoxO3a signaling in mice (Kang et al. 2025). The synthetic myostatin inhibitor, IMB0901, suppresses myostatin promoter activity, signaling, and feedback regulation, reducing muscle wasting in the C26 cancer cachexia model (Liu et al. 2019a).

### Compounds regulating IL-15

Eugenol, a natural phenolic compound from cloves (*Syzygium aromaticum*), controls IL-15 levels in skeletal muscle by activating TRPV1–CaN/NFATc1 signaling, improving endurance capacity and fat browning and suggesting it may act as an exercise mimetic in mice (Huang et al. 2024). Likewise, hydroxylated vitamin D_3_ metabolites, particularly 1,25(OH)_2_D_3_ and 25(OH)D_3_, upregulate IL-15 expression in rodent skeletal muscles in a largely VDR-dependent manner (Ewendt et al. 2025). Conversely, benzoic acid–derived small-molecule IL-15Rα antagonists attenuate systemic IL-15 signaling in immune cells, inhibiting IL-15/IL–15Rα interaction and IL-15-dependent peripheral blood mononuclear cell activation (Krzeczynski et al. 2023).

### Compounds regulating BDNF

Oleuropein, a polyphenolic compound present in olive leaves, exerts neuroprotective effects by regulating the BDNF/CREB/Akt pathway in a rotenone-induced Parkinson’s disease mouse model (Singh et al. 2023). Gastrodin, a phenolic compound extracted from the traditional Chinese herbal medicine, *Gastrodia elata* Blume, upregulates BDNF by inhibiting miR-497, enhancing antioxidant defense and peripheral nerve regeneration in rats (Yongguang et al. 2022). Lactate, a muscle-derived metabolite, increases hippocampal BDNF through SIRT1–PGC1α–FNDC5–TRKB signaling, a pathway linking exercise to BDNF induction in mice (El Hayek et al. 2019). Phloridzin, a plant-derived dihydrochalcone glycoside abundant in apple trees, ameliorates type 2 diabetes–induced depression by upregulating BDNF and mitigating oxidative stress in mice (Kamdi et al. 2021).

### Compounds regulating SPARC

Madecassoside, a triterpenoid saponin derived from *Centella asiatica*, enhances SPARC levels by activating PKC-β and promoting endothelial migration and tube formation, contributing to protection against myocardial infarction in rats (Wang et al. 2025). (−)-Epicatechin, a flavonoid abundant in cocoa that is suggested to be an exercise mimetic in mice (Nogueira et al. 2011), increases SPARC expression in human bone marrow–derived mesenchymal stem cells in parallel with the osteogenic markers, BMP2 and RUNX2, supporting SPARC‐associated osteogenic differentiation (Palma-Lara et al. 2025).

### Compounds regulating FGF21

Maresin 1, an anti-inflammatory lipid mediator, suppresses circulating FGF21 levels and HFD-induced hepatic FGF21 transcription by inhibiting PPARα and counteracts the HFD-induced downregulation of FGF21, FGFR1, and β-Klotho in WAT, whereas in muscle FGF21 remains unchanged and FGFR1 expression increases (Martínez-Fernández et al. 2019). Dimethyl itaconate, a derivative of the tricarboxylic acid cycle, upregulates FGF21 expression in palmitate-treated C2C12 myocytes, alleviating inflammation and insulin resistance (Park et al. 2021). The RORα/γ agonist SR1078 and baicalein, a natural flavone from *Scutellaria baicalensis*, both induce FGF21 expression via the ER-stress marker CHOP in C2C12 myotubes (Hirai et al. 2019b). Sulforaphane, a naturally occurring isothiocyanate derived from cruciferous vegetables, such as broccoli, cabbage, and kale, induces FGF21 and FGFR1 levels in MASLD mice and free fatty acid–treated HepG2 cells, ameliorating hepatic steatosis and inflammation in mice (Wu et al. 2022b). Berberine, a benzylisoquinoline alkaloid, increases FGF21 expression in brown adipose tissue via the AMPK–Bmal1 signaling (Hirai et al. 2019a). Liraglutide, a GLP-1 analog, induces FGF21 secretion in the WAT of type 2 diabetic mice and activates LKB1–AMPK–ACC1 signaling, reducing triglyceride synthesis in macrophages (Zhang et al. 2021). In clinical and experimental myocardial infarction settings, the neprilysin inhibitor (sacubitril) plus the angiotensin II receptor blocker (valsartan) exerts cardioprotective effects by activating FGF21 signaling in mice (Wei et al. 2025). Empagliflozin, a sodium–glucose cotransporter 2 (SGLT-2) inhibitor approved for the treatment of type 2 diabetes and heart failure, has been reported to activate AMPK in skeletal muscle and increase hepatic and circulating FGF21 levels in HFD–induced obese mice, with associated gains in fat utilization, adipose tissue browning, and reduced insulin resistance (Xu et al. 2017). Given the clinical prevalence of SGLT-2 inhibitors and their cardiometabolic benefits, this raises the possibility that FGF21 induction contributes in part to the metabolic effects of this drug class, although causal attribution requires further investigation.

### Compounds regulating apelin

(−)-Epicatechin increases apelin and APJ receptor expression in adipose tissue, accompanied by AMPKα activation and improved lipid metabolism in rats (De Los Santos et al. 2023). A pyrazole-based apelin receptor agonist mimicked the effects of apelin, leading to weight loss, hypophagia, improved glucose metabolism, and reduced liver steatosis in mice (Narayanan et al. 2021). 17β-estradiol, an endogenous estrogen steroid hormone, promotes apelin expression in the right ventricle via ER-α-dependent BMPR2 promoter binding, enhancing right ventricular function and protection in pulmonary arterial hypertension models (Frump et al. 2021).

## Translational and therapeutic implications of targeting myokine pathways

The expanding catalogue of exercise-responsive myokines, together with the growing number of compounds reported to modulate their expression or activity, raises a central translational question: which of these pathways can be pharmacologically leveraged in humans. While preclinical studies demonstrate that elements of muscle endocrine signaling can be modulated independently of contraction, therapeutic translation depends not simply on whether a myokine shows metabolic effects, but on whether its signaling can be engaged with sufficient receptor definition, exposure control, and safety during chronic intervention.

Of these, receptor definition is the clearest determinant of pharmacological tractability. Recombinant proteins and engineered analogues are most suitable when the receptor axis is well-defined, as in the β-Klotho–FGFR1c system for FGF21, where long-acting analogues have advanced to phase 2 and 3 trials in MASH and dyslipidemia (Chui et al. 2024; Jeong et al. 2024). A well-defined G protein-coupled receptor similarly supports small-molecule agonism of the apelin/APJ axis, which has entered first-in-human testing (Winkle et al. 2023). Neutralizing antibodies and ligand traps provide an alternative for receptor-defined ligands that are inhibited rather than replaced, as in the ActRII pathway, though prior experience with ActRII ligand traps also highlights the problem of receptor selectivity, since these agents engage not only myostatin but also activins and GDF11, contributing to off-target vascular and skeletal effects (Campbell et al. 2017). By contrast, pathway-level mimetics, such as AMPK or PPARδ activation, remain inherently partial, reflecting the difficulty of pharmacologically recapitulating the integrated physiology of exercise (Narkar et al. 2008).

More generally, the pharmacokinetics of peptide and protein therapeutics are unfavourable —short half-life and rapid clearance commonly require half-life-extension strategies such as PEGylation, Fc-fusion, or albumin-binding domain conjugation (Andersen et al. 2011; Wu and Sun 2014; Van Witteloostuijn et al. 2016). Therapeutic delivery is a further constraint: the peptidic nature of most myokines restricts administration largely to injectable routes and imposes formulation and stability demands, motivating sustained-release formulations or, where the receptor permits, orally available small-molecule mimetics (Drucker 2020).

Exposure control poses a further challenge because endogenous myokine signaling is typically transient, pulsatile, and tightly coupled to acute metabolic stress, whereas pharmacological interventions often impose sustained systemic exposure. This difference can lead to receptor desensitization, compensatory feedback, or maladaptive remodeling, and helps explain why exercise-mimetic strategies often reproduce only part of the physiological response (Gubert and Hannan 2021). The IL-6 axis illustrates how this exposure mismatch can shape pharmacological strategy. Because its broader inflammatory biology makes chronic IL-6 elevation difficult to deploy as replacement therapy (Rose-John 2012), attention has shifted toward selective modulation of trans-signaling via agents such as olamkicept (Schulte et al. 2022; Zhang et al. 2023).

Safety under chronic intervention is complicated by the pleiotropic nature of myokine signaling. Many myokines are not exclusively muscle-derived in humans, and even established muscle-derived ligands act through receptors expressed across multiple tissues. Achieving adequate tissue specificity may require tissue-biased ligands, targeted conjugates, or local delivery, a limitation most acute for predominantly paracrine factors such as LIF. This multi-organ distribution can increase both therapeutic reach and the risk of mechanism-based toxicity. For example, despite the clear metabolic efficacy of FGF21 analogues, their skeletal effects remain incompletely resolved (Wei et al. 2012). Similarly, although receptor identification has strengthened the mechanistic framework for irisin, concerns regarding context-dependent downstream effects remain (Kim et al. 2018). These examples indicate that translational success depends not only on biological plausibility, but also on defining an appropriate exposure window and target population.

Applied across these requirements, translational readiness varies markedly across the three tiers introduced earlier. Tier 1 axes are at varying stages of clinical translation: FGF21 analogues, ActRII-directed agents, and IL-6 modulation are clinically most advanced, whereas apelin/APJ is pharmacologically tractable but earlier in development. Tier 2 mediators (METRNL, irisin/FNDC5, IL-15, muscle-derived BDNF) await human causal validation, while Tier 3 candidates (GDF11, SPARC, BAIBA, LIF, musclin) remain unresolved at the mediator, target, or assay level for therapeutic prioritization. Overall, only a small subset of myokine pathways currently meets the dual requirement of receptor-level tractability and human causal support.

## Current challenges and future directions

Although individual myokine pathways differ substantially in their translational readiness, several foundational uncertainties limit the field as a whole. Advancing exercise-myokine biology from a catalogue of associations to a platform for therapeutic development will require targeted progress in four interrelated areas: receptor and target definition, analytical rigor, human causal inference, and context-resolved biology.

The first challenge is receptor and target definition. For many candidate myokines, receptor identity, tissue specificity, and proximal signaling remain incomplete, making it difficult to distinguish causal mediators from secondary correlates and to verify target engagement in vivo. Recent work on integrin αVβ5 for irisin and KIT for METRNL illustrates how receptor assignment sharpens mechanistic and translational interpretation, but comparable receptor-level resolution is still lacking for many factors reviewed here. A central priority is therefore systematic receptor deorphanization using orthogonal approaches, including CRISPR activation–based surface receptor screening, high-avidity ligand-binding enrichment, and structure-guided prediction pipelines, followed by quantitative validation of binding, receptor dependency, and in vivo signaling (Siepe et al. 2022; Banhos Danneskiold-Samsoe et al. 2024; Yang et al. 2024).

A second challenge is analytical rigor. For several widely studied myokines, uncertainty in measurement remains large enough to confound biological interpretation, as exemplified by the antibody-based assay controversy for irisin, myostatin-GDF11 cross-reactivity, and the strong sensitivity of circulating BDNF to sample handling. These cases share a common implication. More rigorous standardization of pre-analytical handling, assay selectivity, and orthogonal validation should therefore be treated as a prerequisite rather than a refinement step for any myokine proposed as a biomarker or therapeutic target.

A third challenge is that human evidence remains largely correlative. Most studies still rely on pre–post exercise comparisons without establishing tissue of origin, necessity, or target engagement, and this limitation is compounded by heterogeneity in exercise protocols, sampling time points, and participant characteristics (Bettariga et al. 2024; Ringleb et al. 2024). Future human studies should place greater emphasis on source attribution and causal inference, using arteriovenous balance measurements, matched tissue–plasma analyses, and target engagement biomarkers where feasible. Receptor blockade trials in humans, such as those that have clarified the role of IL-6 in exercise-induced visceral fat loss, provide a template for how endogenous myokine signaling can be interrogated causally rather than associatively.

The fourth challenge is insufficient attention to biological context and tissue resolution. Myokine signaling is strongly influenced by age, sex, hormonal status, and metabolic state, yet many studies continue to rely on small, relatively homogeneous cohorts. Emerging evidence indicates that myokine regulatory networks are shaped by sex hormones and genetic background (Velez et al. 2022), and that aging can reduce receptor responsiveness even when ligand production is preserved (Sakamoto et al. 2024). In parallel, the cellular origin of many myokines within muscle remains incompletely resolved. Because skeletal muscle contains diverse myofiber, stromal, vascular, and immune populations, whole-muscle measurements can obscure the actual source of a secreted factor. Single-cell and single-nucleus atlases, spatial transcriptomics, and fiber-type–resolved proteomics now provide the tools to address this problem more directly (Petrany et al. 2020; Murgia et al. 2021; Lai et al. 2024).

Looking ahead, the next phase of the field will depend on resolving these foundational uncertainties. Priorities include receptor-level definition, assay standardization, human source attribution, context-stratified study design, and experimental systems that better link cellular secretion to target engagement and physiological function. Advanced human-relevant models, including electrically stimulated myotubes and contractile 3D muscle systems, may help bridge this gap (Evers-Van Gogh et al. 2015; Sugimoto et al. 2022), but carefully designed perturbation-based human studies will remain essential for establishing causality and defining the true therapeutic relevance of individual myokine pathways.

Beyond these specific methodological priorities, a broader caveat applies to the entire framework outlined above. Although this review is organized around individual myokines, exercise adaptation is not reducible to any single secretome. Contracting skeletal muscle simultaneously releases peptides, lipid mediators, exosomal microRNAs, and small-molecule metabolites, and these signals act in parallel with mechanical, neural, endocrine, vascular, immune, and microbiome-derived inputs to produce integrated systemic adaptation. Two implications follow. First, beyond the exposure-mismatch issues discussed earlier, any single myokine-based intervention will engage only a fraction of the broader signaling architecture and cannot, in itself, recapitulate exercise as an integrative phenomenon. Second, advancing the field will increasingly require integrative approaches — including multi-omic profiling, network-level analysis, and combinatorial perturbation — that move beyond the cataloguing of individual factors toward a mechanistic account of exercise biology as an integrated physiological response.

## Conclusions

This review synthesizes recent advances in exercise-induced myokines—their physiological roles in metabolic regulation, the natural and synthetic compounds reported to modulate their expression, secretion, or activity, and the opportunities and constraints of targeting these pathways pharmacologically. The central conceptual advance of this updated synthesis is that myokines are not a uniform group of exercise factors, but a biologically heterogeneous network that varies in mechanistic definition, source attribution, and translational readiness. Among these axes, only a small subset of myokine pathways—most notably FGF21 analogues, ActRII-directed agents, IL-6 modulation, and apelin/APJ—currently sit at the intersection of validated target biology and credible human causal data. Collectively, these findings reinforce the view that myokines are important mediators of muscle–organ crosstalk, with relevance to metabolic disease, sarcopenia, and healthy aging, and provide a plausible basis for exercise-inspired therapeutic development.
